# Structural Gating Enhances Long-Distance Light-Driven Interfacial Electron Transfer

**DOI:** 10.1021/acscentsci.4c01106

**Published:** 2024-11-11

**Authors:** Quentin R. Loague, Marzieh Heidari, Hayden J. Mann, Evgeny O. Danilov, Felix N. Castellano, Elena Galoppini, Gerald J. Meyer

**Affiliations:** † Department of Chemistry, University of North Carolina at Chapel Hill, Chapel Hill, North Carolina 27599, United States; ‡ Department of Chemistry, Rutgers University, 73 Warren Street, Newark, New Jersey 07102, United States; § Department of Chemistry, North Carolina State University, Raleigh, North Carolina 27695-8204, United States

## Abstract

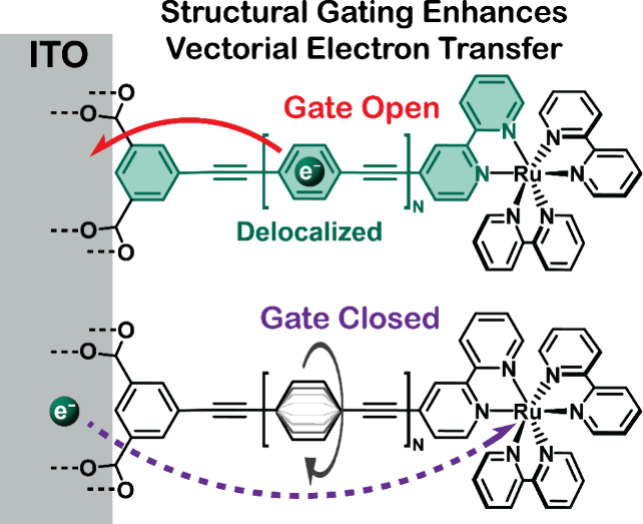

Structural gating provides a molecular means to transfer electrons preferentially in one desired vectorial direction, a behavior needed for applications in artificial photosynthesis. At the interfaces utilized herein, visible-light absorption by a transition metal complex *opens* a “structural gate” by planarization of otherwise rotating phenyl rings in *p-*phenylene ethynylene (PE) bridge units. Planarization provides a conjugated pathway for electron flow toward a conductive oxide surface. Interfacial electron transfer to the oxide restores rotation and *closes* the gate to the unwanted recombination reaction. This structural gating results in nearly quantitative long-distance (>20 Å) interfacial electron transfer that occurs ∼1000 times faster than transfer in the opposite direction. A comparative kinetic study of these complexes with those that contain ionic bridge units, without gating function, as a function of the applied potential and hence −Δ*G*° provided a physical basis for the structural gating. A small distance-dependent reorganization energy with weak electronic coupling underlies the success of this gate that enables efficient long-distance electron transfer and slow recombination.

## Abstract 2

A light-triggered structural gate provides a conjugated pathway for efficient long-distance vectorial electron transfer to a conductive oxide and inhibits transfer from the oxide.

## Introduction

Molecular approaches to solar energy conversion generally require the preferential transfer of electrons in one specific direction. This has been termed “vectorial” electron transfer, encompassing both the mathematical definition of transfer from an origin to a particular location and the kinetic definition of a velocity with a known magnitude and direction.^1−5^ Natural^6−9^ and artificial^10−13^ photosynthetic assemblies accomplish vectorial transfer through free energy gradients with spatially arranged redox centers that direct electrons toward catalytic sites while providing a thermodynamic penalty for transfer in the opposite direction.^14^ An alternative approach is to gate between two distinct molecular geometries: one that promotes vectorial electron transfer and the other that inhibits transfer. Such structural, or conformational, gating has been implicated in natural photosynthesis and is relevant to the celebrated “entactic state” hypothesis in proteins and model systems.^15−19^ Herein, structural gating is utilized to optimize the quantum yield for light-driven long-distance electron transfer from a photoexcited transition metal complex to a conductive oxide material with slow recombination.^20−26^

Campagna and co-workers noted the impact of aromatic bridge units on intramolecular transfer in artificial photosynthetic assemblies.^27^ Later studies showed the generality of the finding^28^ and demonstrated how rate constants could be controlled synthetically with bridge substituents that tune the degree of conjugation.^24,29^ More recently and of more relevance to this study, Spettel and Damrauer realized vectorial electron transfer^30^ through a rapid ∼2 ps conformational change at a semiconducting TiO_2_ interface.^24,31,32^ The unwanted reverse reaction was slowed by a factor of 12 relative to a model complex without the structural gating motif.^30^ Kinetic evidence for structural gating at TiO_2_ interfaces was also reported in a comparative study where the degree of planarization between two aromatic groups was controlled.^33^ In addition, computational and experimental measurements support the presence of structural gates with aromatic bridge units.^34−36^ These advances in vectorial electron transfer are noteworthy, as structural gating does not necessarily require the free energy losses inherent to electron hopping down free energy gradients.^14^

**Scheme 1:**
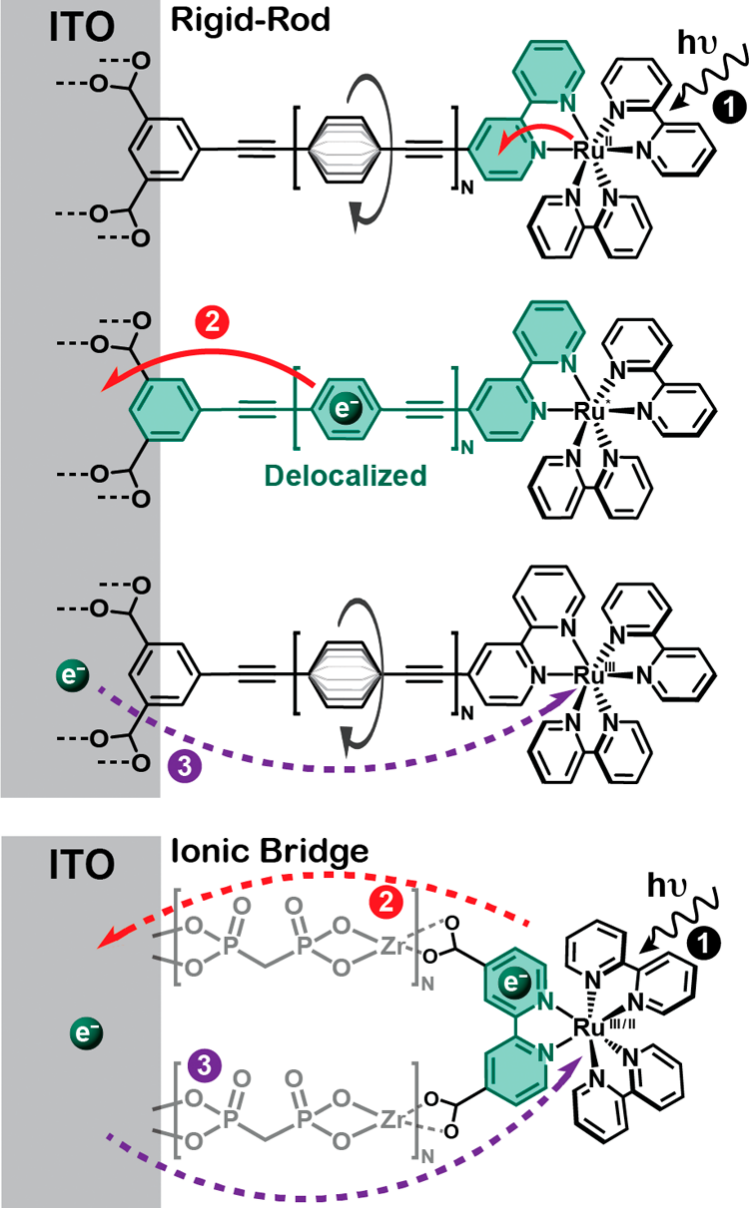
Proposed Light-Initiated Structural Gating Mechanism for *p*-Phenylene Ethynylene (PE) Bridge Units That Is Absent for Ionic Bridges Light absorption generates a metal-to-ligand charge-transfer (MLCT) excited state (1). For rigid-rod complexes, this initiates planarization of the PE bridge units to provide a conjugated pathway for electron transfer to the oxide, termed excited-state injection (2). Ionic bridges with methylenediphosphonic acid–Zr^4+^ layers, termed ionic-bridge complexes and herein abbreviated as ITO|−(X)_*N*_–RuC (*N* = 0, 1, 2), do not possess this gating function. Charge recombination of the injected electron with the oxidized metal center (3) was the subject of recent studies.^45,46^

Here we report excited-state electron transfer from a ruthenium polypyridyl excited state to the conductive oxide material tin-doped indium oxide (ITO). Two classes of complexes were utilized to position the excited state at different locations within the electric double layer (Scheme 1).^37−41^ Rigid-rod complexes that contained zero, one, or two *p-*phenylene ethynylene (PE) bridge units were competent for structural gating. Ionic bridges based on Zr(IV) and phosphonate provided insulating bridges that were incompetent for structural gating. The basis for structural gating is as follows. In the ground electronic state of the rigid-rod complexes, the phenyl rings in adjacent bridge units are unconjugated. Metal-to-ligand charge-transfer (MLCT) excitation (1) results in the formal reduction of the bipyridine ligand, which initiates planarization of these phenyl rings,^31^ providing an open-gate, delocalized electron transfer pathway to the carboxylate groups and the ITO surface. This open-gate excited state has been termed the rigid-rod or RR state. Interfacial electron transfer to the ITO (2), termed excited-state electron injection, restores rotation of the bridge phenyl rings and closes the gate for the unwanted reverse reaction to the oxidized Ru(III) metal center (3). The reorganization energies, λ, and electronic couplings, *H*_ab_, are expected to be sensitive to these structural changes. Indeed, Marcus–Gerischer analysis of the kinetic data allowed extraction of these parameters that are needed to develop predictive models.^42−44^ Previous comparative studies showed that λ and *H*_ab_ were slightly smaller when carboxylic acid anchoring groups were utilized (λ = 0.40 eV and *H*_ab_ = 0.84 cm^–1^) relative to phosphonic acids (λ = 0.52 eV and *H*_ab_ = 0.94 cm^–1^), thereby demonstrating the utility of the analysis.^46^ In the present study, Marcus–Gerischer analysis revealed that a small reorganization energy was responsible for the near quantitative yield of long-distance (>20 Å) excited-state electron transfer through PE bridge units.

## Results

**Figure 1:**
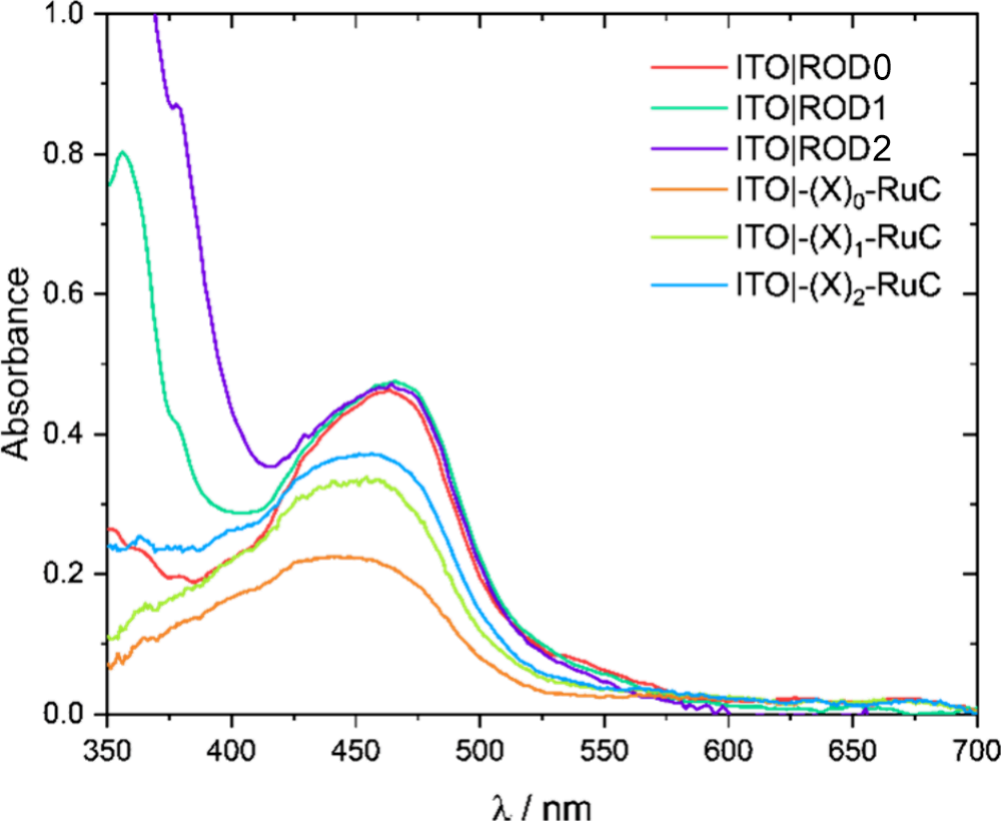
Visible absorption spectra of the indicated ITO|ROD and ITO|−(X)_*N*_–RuC materials measured in 0.1 M LiClO_4_/CH_3_CN.

Visible absorption spectra of the rigid-rod and ionic-bridge complexes anchored to ITO and immersed in a 0.1 M LiClO_4_/CH_3_CN electrolyte are shown in Figure 1. The MLCT absorption centered near 460 nm was insensitive to the number of bridge units. The enhanced absorption at wavelengths below 425 nm was due to light absorption by the PE bridge units. The surface coverages, determined from visible absorption measurements with a modified Beer’s law,^47^ were in the (2–3) × 10^–8^ mol/cm^2^ range, which is typical for transition metal complexes anchored to mesoporous thin films.^48^ The formal reduction potentials *E*°′(Ru^III/II^) of the rigid-rod and ionic-bridge complexes have been reported previously and were found to be insensitive to the bridge identity (Table 1).^45,46,49^

**Table 1:** Electrochemical and Spectroscopic Properties of Sensitized ITO Films

	*E*°′(Ru^III/II^) (V vs NHE)	*E*°′*(Ru^III/II*^) (V vs NHE)	PL_max_ (nm) [τ (μs)]^a^	λ_abs_ (nm) [ε (M^–1^ cm^–1^)]^b^	Γ (mol/cm^2^)
ITO|ROD0	1.52	–0.40	646 [6.9]	462 [1.6 × 10^4^]	2.9 × 10^–8^
ITO|ROD1	1.52	–0.40	646 [7.4]	465 [2.0 × 10^4^]	2.4 × 10^–8^
ITO|ROD2	1.51	–0.41	646 [7.7]	465 [2.0 × 10^4^]	2.4 × 10^–8^
ITO|−(X)_0_–RuC	1.48	–0.44	644	460 [1.2 × 10^4^]	1.8 × 10^–8^
ITO|−(X)_1_–RuC	1.48	–0.44	644	460 [1.2 × 10^4^]	2.8 × 10^–8^
ITO|−(X)_2_–RuC	1.48	–0.44	644	460 [1.2 × 10^4^]	3.1 × 10^–8^

a Photoluminescence data were measured for the complexes anchored to insulating ZrO_2_ films.

b Extinction coefficients were taken from refs (49) and (50).

**Figure 2:**
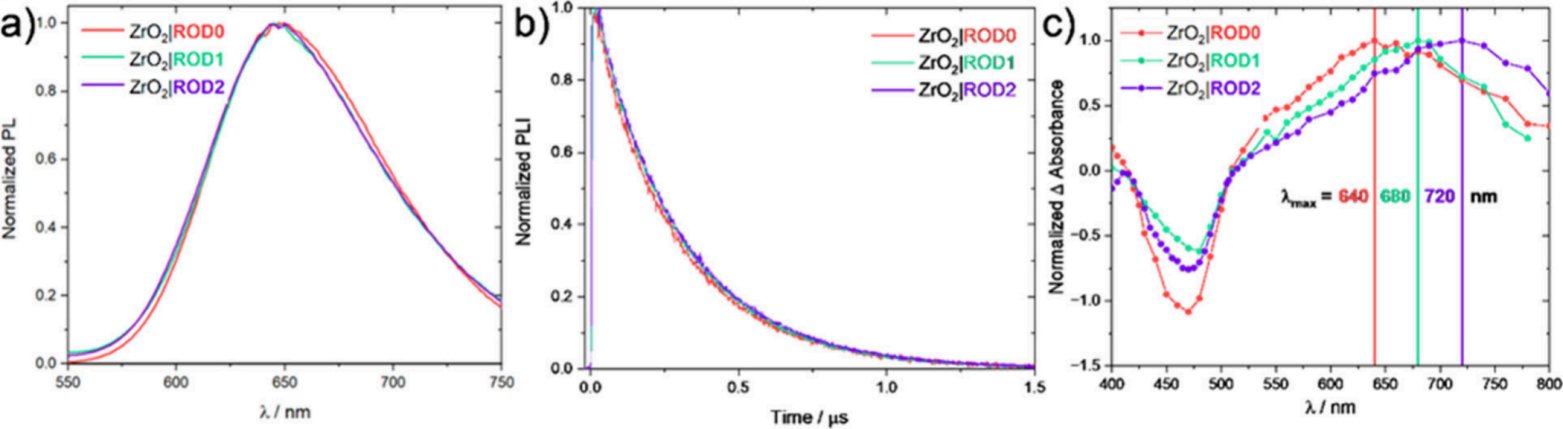
(a) Normalized photoluminescence spectra of ZrO_2_|ROD*x* in CH_3_CN. (b) Time-resolved photoluminescence intensity decay kinetics (λ_obs_ = 650 nm) of ZrO_2_|ROD*x* in CH_3_CN. (c) Transient absorption difference spectra (100 ns after pulsed excitation) of ZrO_2_|ROD*x* in 0.1 M LiClO_4_/CH_3_CN (λ_exc_ = 488 nm, 6 ns fwhm).

The rigid-rod complexes displayed room temperature photoluminescence (PL) in fluid solution and when anchored to insulating ZrO_2_ thin films immersed in 0.1 M LiClO_4_/CH_3_CN. The PL maxima were insensitive to the number of rigid-rod linkers (Figure 2a and Table 1), with only a small difference in the spectra for ROD0. Time-resolved PL decays measured after pulsed light excitation were well-described by a first-order kinetic model when anchored to ZrO_2_ at room temperature (Figure 2b) and at 77 K in frozen organic glass (MeTHF, Figure S1). The excited-state lifetime, τ, increased slightly as the number of PE bridge units increased (Table 1). The lifetimes were in good agreement with those measured by transient absorption spectroscopy. The excited-state reduction potential, *E*°′*(Ru^III/II*^), was calculated from eq 1:
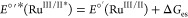
1where the Gibbs free energy stored in the excited state, Δ*G*_es_, was extracted from the peak of the room-temperature photoluminescence spectra (Table 1).^51^

Figure 2c shows the transient absorption difference spectra of the three rigid-rod complexes anchored to ZrO_2_ thin films immersed in 0.1 M LiClO_4_/CH_3_CN. Normalized spectra measured at different delay times were the same within experimental error. Clean isosbestic points were present and consistent with excited-state decay directly to the ground state. The positive absorption band below 400 nm and bleach at ∼450 nm are qualitatively similar to related MLCT excited states.^52^ In contrast to standard complexes like [Ru(bpy)_3_]^2+^, the intense positive absorption band in the 600–800 nm region shifted to lower energy with an increasing number of PE units, implying delocalization of the excited state across the PE bridge.^53−55^ This state is assigned as a rigid-rod excited state (“RR state”) wherein the excited electron is delocalized on the PE bridge units and the metal center is in the formal oxidation state of III.

**Figure 3:**
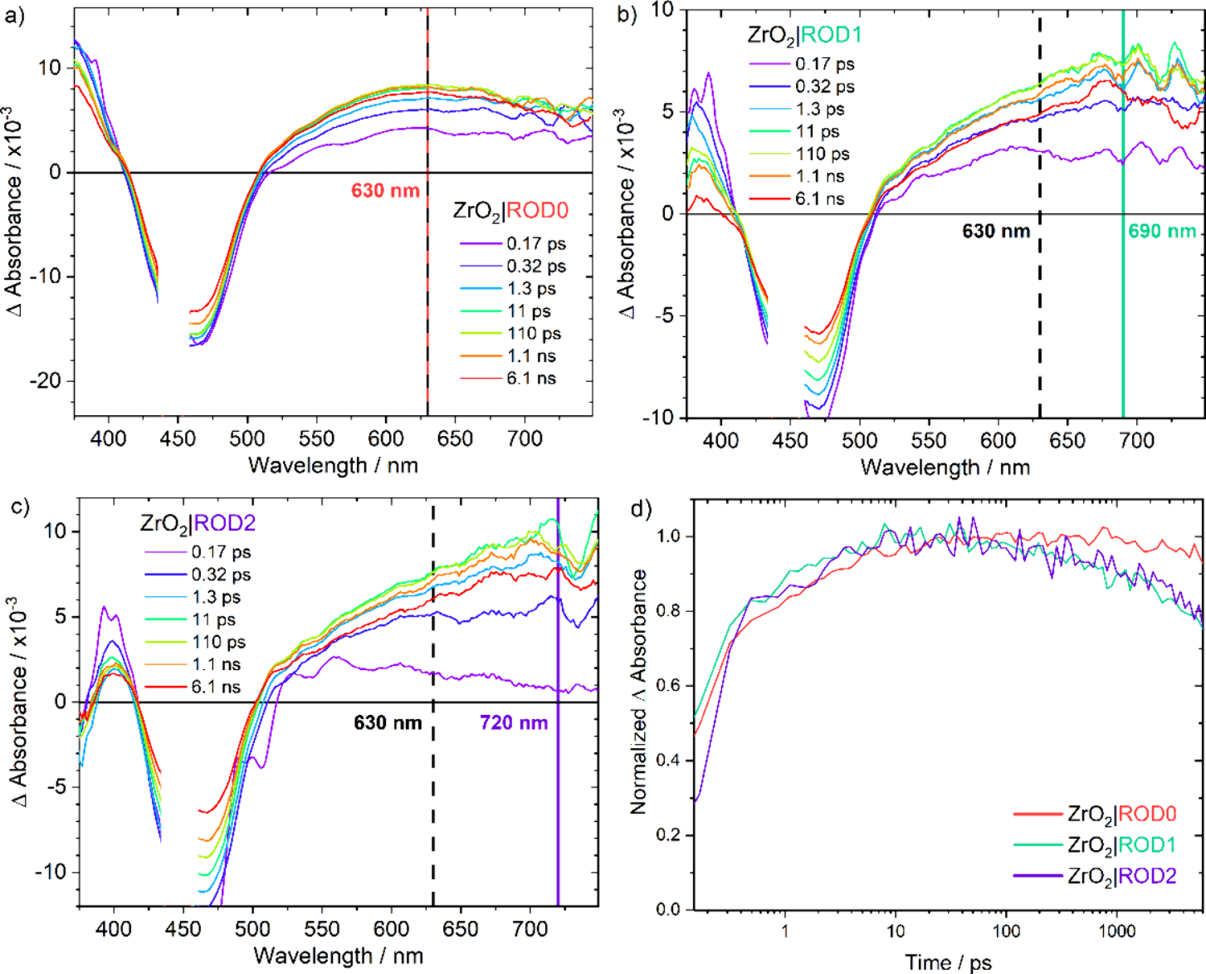
(a–c) Transient absorption difference spectra measured from 160 fs to 6 ns after pulsed 440 nm laser excitation for (a) ZrO_2_|ROD0, (b) ZrO_2_|ROD1, and (c) ZrO_2_|ROD2 in 0.1 M LiClO_4_/CH_3_CN. (d) Transient absorption kinetics for all three rigid-rod complexes at (a) 630 nm, (b) 690 nm, and (c) 720 nm.

Ultrafast transient absorption spectroscopic measurements were performed to gain additional insight into the excited-state behavior. Figure 3 presents the time-resolved absorption difference spectra measured between 170 fs and 6.1 ns after pulsed 440 nm light excitation (∼100 fs) of the indicated rigid-rod complex anchored to insulating ZrO_2_. The difference spectrum recorded at 170 fs for ZrO_2_|ROD2 was typical of an MLCT excited state with a positive absorption near 390 nm, a bleach of the MLCT absorption near 460 nm, and a weak broad absorbance in the red region. The absorbance at ∼390 nm has been assigned as a π* → π absorption of the formally reduced diimine ligand.^52^ Over the next 170 fs, the amplitude of this band and the MLCT bleach decreased with a dramatic growth of the broad band in the red region (τ = 830 fs^–1^). These time-dependent spectral changes were most pronounced for ZrO_2_|ROD2 and the smallest for ZrO_2_|ROD0. For example, the broad absorption in the 600–800 nm region increased and saturated at twice the 170 fs amplitude for ZrO_2_|ROD0, while that for ZrO_2_|ROD1 increased 4-fold and that for ZrO_2_|ROD2 about 10-fold. The spectra recorded at ∼6 ns were in good agreement with those measured on nanosecond and longer time scales.^45,49^ For all of the rigid-rod complexes, the intermediate electronic state was formed in less than 10 ps and relaxed cleanly to the ground state on a microsecond time scale.

**Figure 4:**
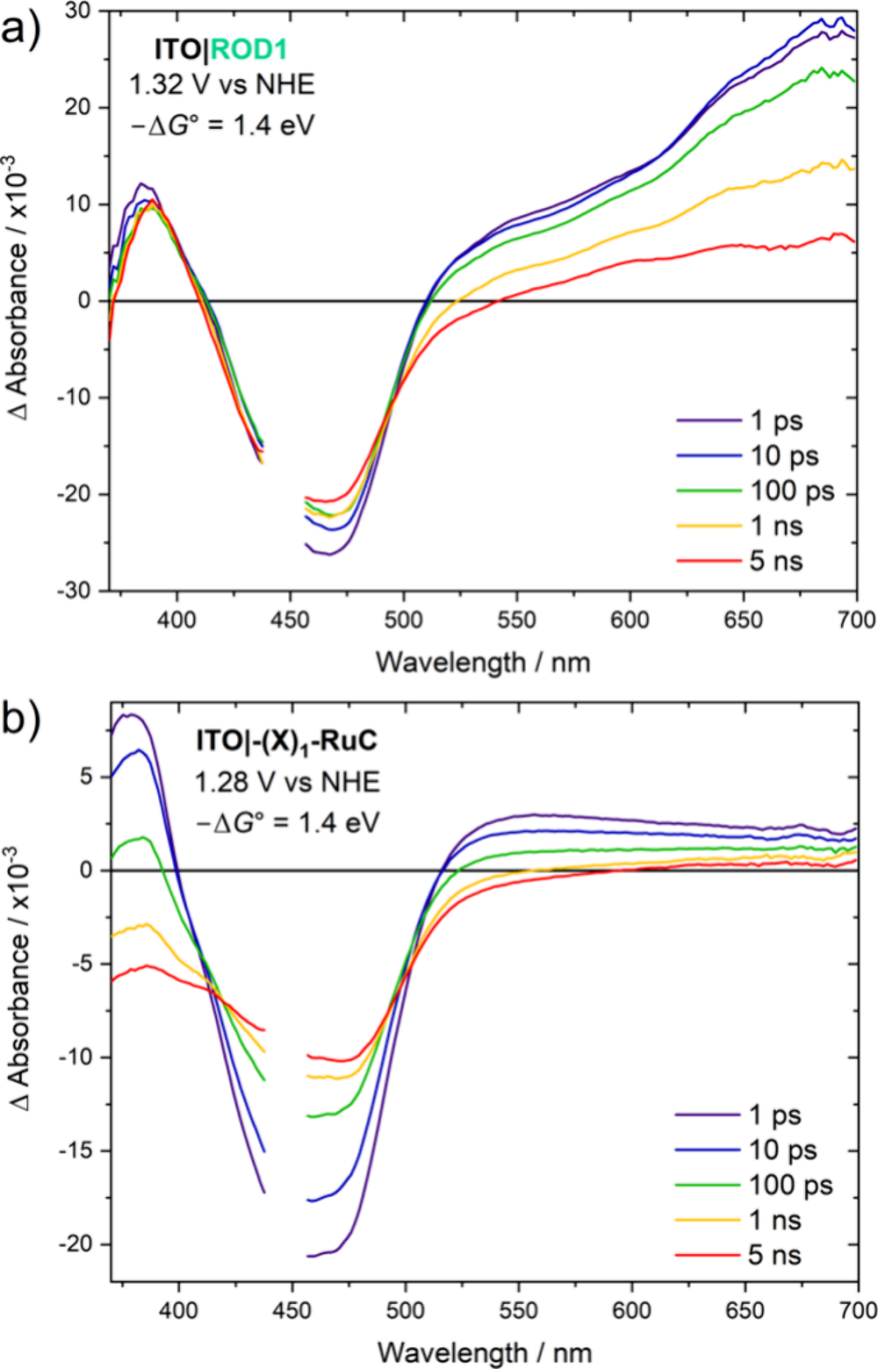
Transient absorption difference spectra measured at the indicated delay times after pulsed 440 nm laser excitation (150 fs) of (a) ITO|ROD1 and (b) ITO|−(X)_1_–RuC in 0.1 M LiClO_4_/CH_3_CN with the indicated applied potential that corresponds to −Δ*G°* = 1.4 eV for excited-state injection into ITO. Spectra for other samples are provided in Figure S2.

The kinetics for excited-state electron transfer to ITO was quantified by subpicosecond transient absorption spectroscopy as a function of the applied potential, *V*_app_, in a standard three-electrode cell in 0.1 M LiClO_4_/CH_3_CN. Figure 4 shows absorption difference spectra measured at the indicated delay times after pulsed 440 nm laser excitation for ITO|ROD1 and ITO|−(X)_1_–RuC at the indicated *V*_app_, corresponding to a −1.4 eV driving force for the excited-state electron transfer reactions ITO|Ru^II*^ → ITO(e^–^)|Ru^III^. The spectral data for the other complexes are listed in Figure S2. At a 1 ps delay time, the spectra corresponded to the RR state for ITO|ROD1 and to the MLCT excited state for ITO|−(X)_1_–RuC. This assignment was based on the excited-state difference spectra of the complexes anchored to an insulating ZrO_2_ thin film, Figure 3. These spectra continued to evolve with time and were predominantly charge-separated states by 5 ns, comprised of an injected electron and an oxidized Ru complex. Assignment of the charge-separated states were confirmed by spectral comparisons to spectroelectrochemical data of the complexes upon oxidation of Ru^II^ to Ru^III^.^45,46^

**Figure 5:**
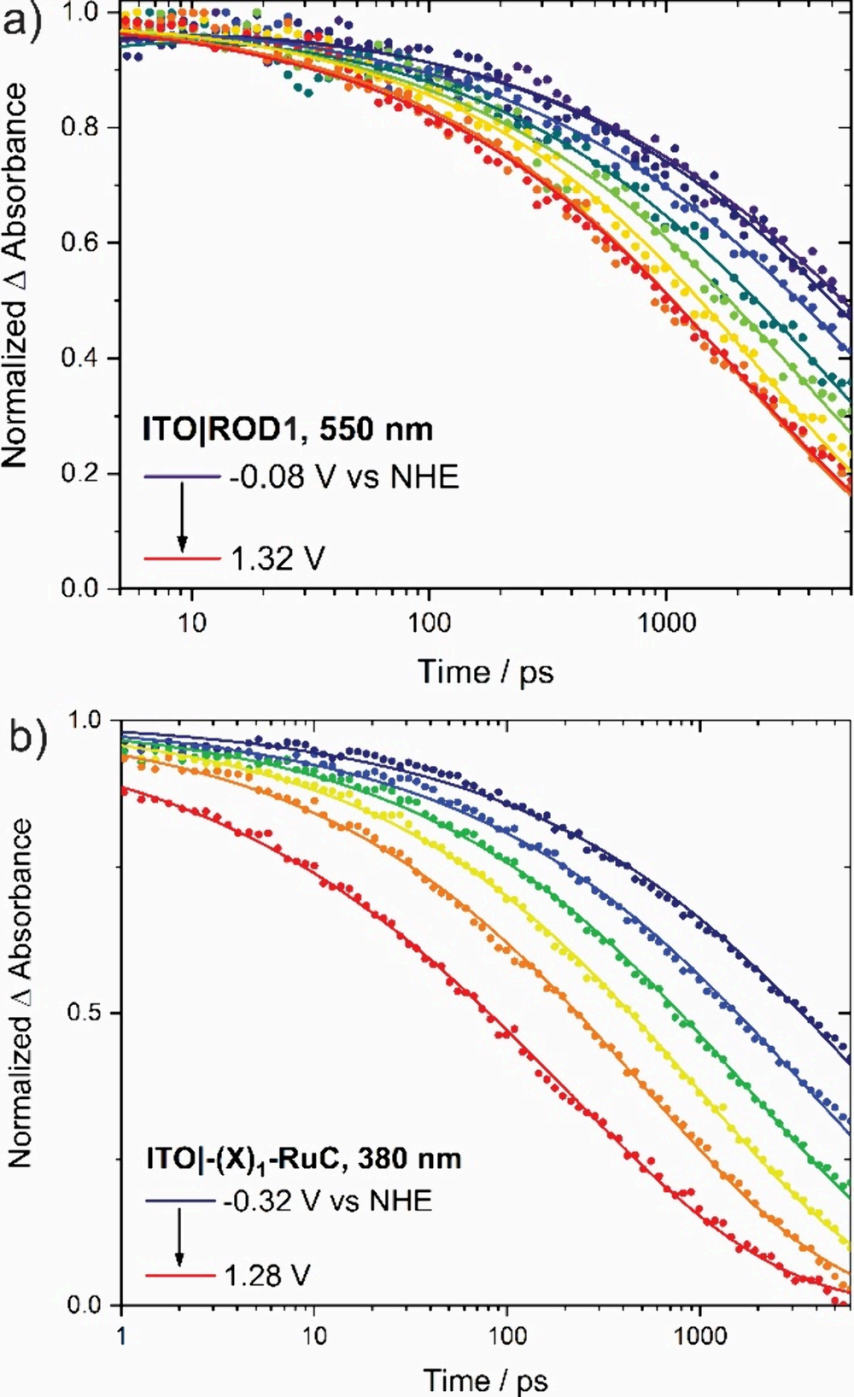
Normalized absorption changes monitored after pulsed excitation (440 nm, 150 fs fwhm) of (a) ITO|ROD1 and (b) ITO|−(X)_1_–RuC in 0.1 M LiClO_4_/CH_3_CN over the applied potential ranges indicated. Transient absorption kinetics for other samples are provided in Figure S4. Further information about global analysis and normalization techniques are provided in the Supporting Information.

Figure 5 shows the absorption changes monitored at 380 nm for ITO|−(X)_1_–RuC and 550 nm for ITO|ROD1 after pulsed 440 nm light excitation at selected applied potentials over a 10 to 6000 ps window. The applied-potential-dependent kinetic data for the other complexes are given in Figure S4. The observation wavelengths were arbitrarily chosen to be where the signal amplitude (Δ*A*) was the largest. These data were normalized to unity and scaled such that quantitative electron transfer corresponded to a final Δ*A* of zero. The non-normalized data are shown in Figure S3. The kinetic data reveal that the injection rate increased for both complexes as the applied potential was made more positive. Further, at any applied potential, the yield of electron transfer products was larger for (ITO|ROD1) than for (ITO|−(X)_1_–RuC). The kinetic data were nonexponential and well-modeled by the Kohlrausch–Williams–Watts (KWW) function (eq 2), which was previously used to model interfacial electron transfer at nanoparticle surfaces:^56−59^
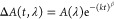
2where β is inversely related to the width of an underlying Levy distribution of rate constants, through which average rate constants *k̅* were determined from the first moment (eq 3):
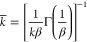
3The kinetic data were analyzed globally across all wavelengths and provided the average rate constants *k̅* given in Table S1.

**Figure 6:**
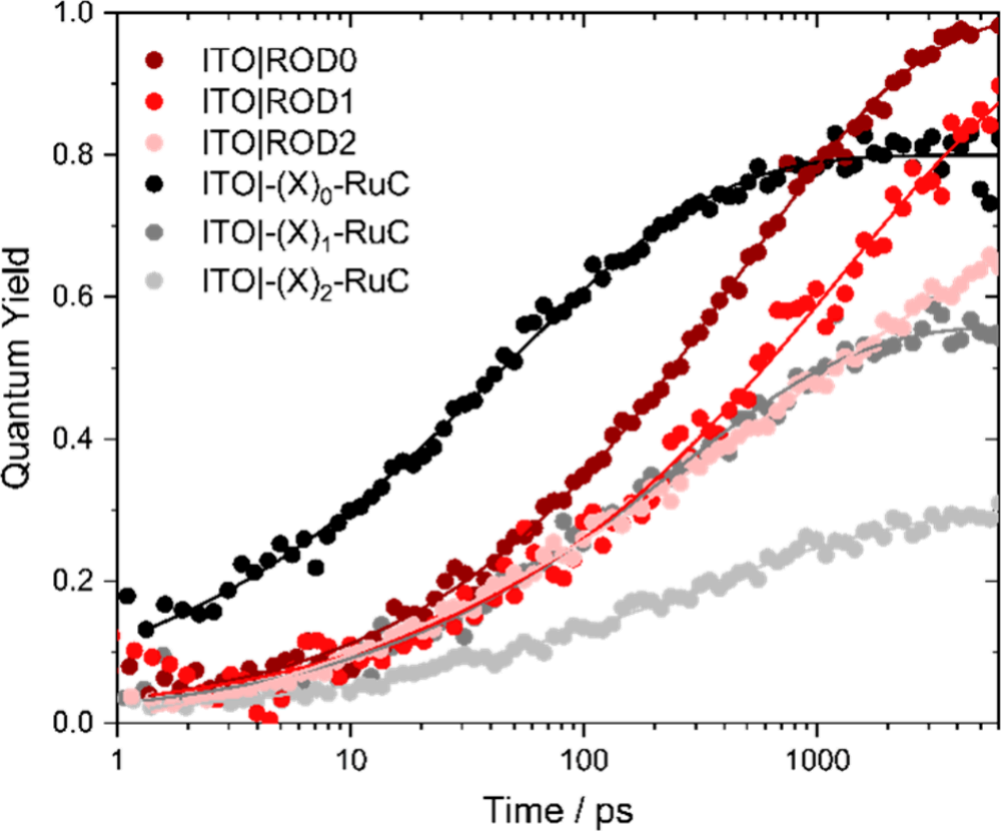
Quantum yield for excited-state injection monitored after pulsed excitation (440 nm, 150 fs fwhm) in 0.1 M LiClO_4_/CH_3_CN with an applied potential that corresponds to −Δ*G°* = 1.4 eV. Representative KWW global fits (solid lines) are overlaid onto the indicated single-wavelength kinetic traces (points).

Figure 6 shows the quantum yield for interfacial electron transfer as a function of time measured at a constant driving force for injection, −Δ*G°* = 1.4 eV. The data reveal that injection was fastest for ITO|−(X)_0_–RuC, followed by the ionic-bridge complexes with *N* = 1 *>N* = 2, with injection quantum yields decreasing in the same order (as determined by comparative actinometry).^60^ The injection yield for ITO|−(X)_2_–RuC was only 0.3. Injection for the rigid-rod complexes was slower than for the ionic-bridge complexes and decreased in the order ROD0 > ROD1 > ROD2. However, the quantum yield remained near unity for ROD0 and ROD1 and decreased only slightly for ROD2 (Table 2). Overlaid on these data are fits to the KWW function, from which the quantum yield values were determined. Additional details of this kinetic analysis are given in the Supporting Information.

**Table 2:** Marcus–Gerischer Analysis of Interfacial Charge Separation Kinetic Data as a Function of Electron Transfer Distance *R*

		injection			recombination^a^	
	*R* (Å)	ϕ_inj_	λ (eV)	*H*_ab_ (cm^**–**1^)	λ (eV)	*H*_ab_ (cm^**–**1^)
ITO|ROD0	14	∼1	0.87	6.8	0.61	0.54
ITO|ROD1	21	∼1	1.06	3.7	0.65	0.21
ITO|ROD2	28	0.86	1.01	2.8	0.80	0.11
ITO|-(X)_0_-RuC	8	0.80	0.86	19	0.57	0.84
ITO|-(X)_1_-RuC	18	0.56	1.30	9.4	0.87	0.16
ITO|-(X)_2_-RuC	26	0.30	>1.60	7.3	0.93	0.12

a Reorganization energies for recombination were taken from refs (45) and (46).

## Discussion

**Figure 7:**
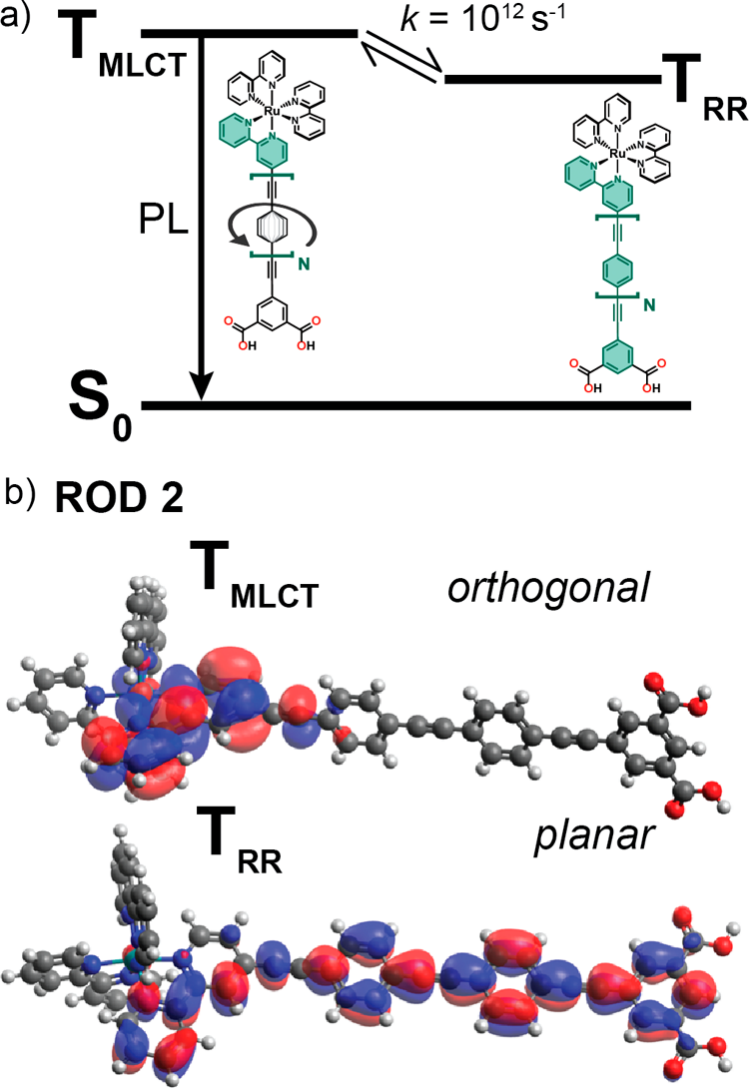
(a) Simplified Jablonski diagram illustrating the two excited states found for the rigid-rod complexes, T_MLCT_ and T_RR_. (b) Representative molecular orbitals for the T_MLCT_ (orthogonal) and T_RR_ (planar) states determined from TD-DFT. Complete molecular orbitals (Figures S8 and S9) with experimental details are provided in the Supporting Information.

Excited-state electron transfer from transition metal complexes bearing phenylene ethynylene (PE) or ionic bridge units to the conductive oxide ITO was kinetically resolved and measured as a function of the thermodynamic driving force. This research complements a previous study of the unwanted charge recombination reaction of the electron in the ITO with the oxidized form of these same complexes.^45^ Such comparative data are rare in the vast literature on dye sensitization and allow a complete description of the charge separation and recombination reactions across conductive oxide interfaces.^44^ An unexpected finding was the presence of two long-lived electronic excited states for the rigid-rod complexes: a photoluminescent metal-to-ligand charge-transfer (MLCT) excited state and a rigid-rod (RR) excited state, as shown in Figure 7. The interactions of these two states underlie the proposed *excited-state gating mechanism* that enables nearly quantitative excited-state electron transfer with slow recombination at distances greater than 20 Å. Indeed, the “open gate” corresponds to the coplanar phenyl bridge structure that provides a pathway for efficient long-distance vectorial excited-state interfacial electron transfer.

The free energy dependence of the excited-state electron transfer kinetic data was consistent with expectations of Marcus–Gerischer theory and provided quantitative values of the reorganization energy λ and the electronic coupling matrix element *H*_ab_. These values are contrasted with those for the unwanted charge recombination reaction below. Nearly quantitative long-distance electron transfer resulted from a relatively small reorganization energy in the planar and conjugated orientation of the PE bridge units. Surprisingly, *H*_ab_ was smaller than that measured for the corresponding ionic-bridge complexes.^59^ Curiously, λ for injection was larger than that for recombination, behavior at odds with the accepted dogma for interfacial electron transfer in dye-sensitized solar cells.^59^ Taken together, the data indicate that the reorganization energy is a far more significant contributor to structural gating than is electronic coupling at these interfaces.

### Excited-State Gating Mechanism

An interesting aspect of the gating mechanism is the presence of two electronic excited states for the rigid-rod complexes, as shown in Figure 7. An excited-state absorption band whose maximum wavelength increased with the number of PE bridge units was evident. In contrast, the photoluminescence (PL) spectra were insensitive to the number of bridge units and were instead typical of an MLCT excited state. Ultrafast transient absorption studies revealed that the MLCT excited state evolved into the second excited state on a 10^–12^ s time scale. Indeed, the spectra measured 10 ps after excitation were the same as those measured on nanosecond and longer time scales and was assigned to an RR state comprising an excited electron delocalized over coplanar PE bridge units and an oxidized Ru(III) center. The lifetimes measured by transient absorption and photoluminescence were the same within experimental error, indicating a quasi-equilibrium between the MLCT and RR states. The rapid picosecond MLCT → RR relaxation indicates a small kinetic barrier between these states. For the ionic-bridge complexes, the spectral data were consistent with the formation of a single localized MLCT excited state. We note that a transient infrared study of photoexcited PE-bridged donor–acceptor compounds also revealed the presence of a long-lived nonemissive charge transfer state.^61^ The dihedral angle between phenyl rings on adjacent bridge units has also been correlated with the photophysical properties of PE-based polymers.^62,63^

The electronic structure of the RR and MLCT excited states was probed computationally. Each was optimized in the gas phase for both singlet and triplet electron configurations with dihedral angles of 0° and 90° between the PE bridge units and the pyridine ring in the bipyridine ligand. Figure 7b reveals minimal charge density on the PE bridge units in the 90° orientation of the MLCT excited state. In contrast, planarization results in significant conjugation across the PE bridge units to the carboxylic acid binding groups. A cumulene-type structure is evident in the planarized state, where the ethyne bond length decreases and the phenyl C–C bond lengths increase, consistent with previous transient infrared studies of 1,4-bis(phenylethynyl)benzene compounds.^64,65^

**Figure 8:**
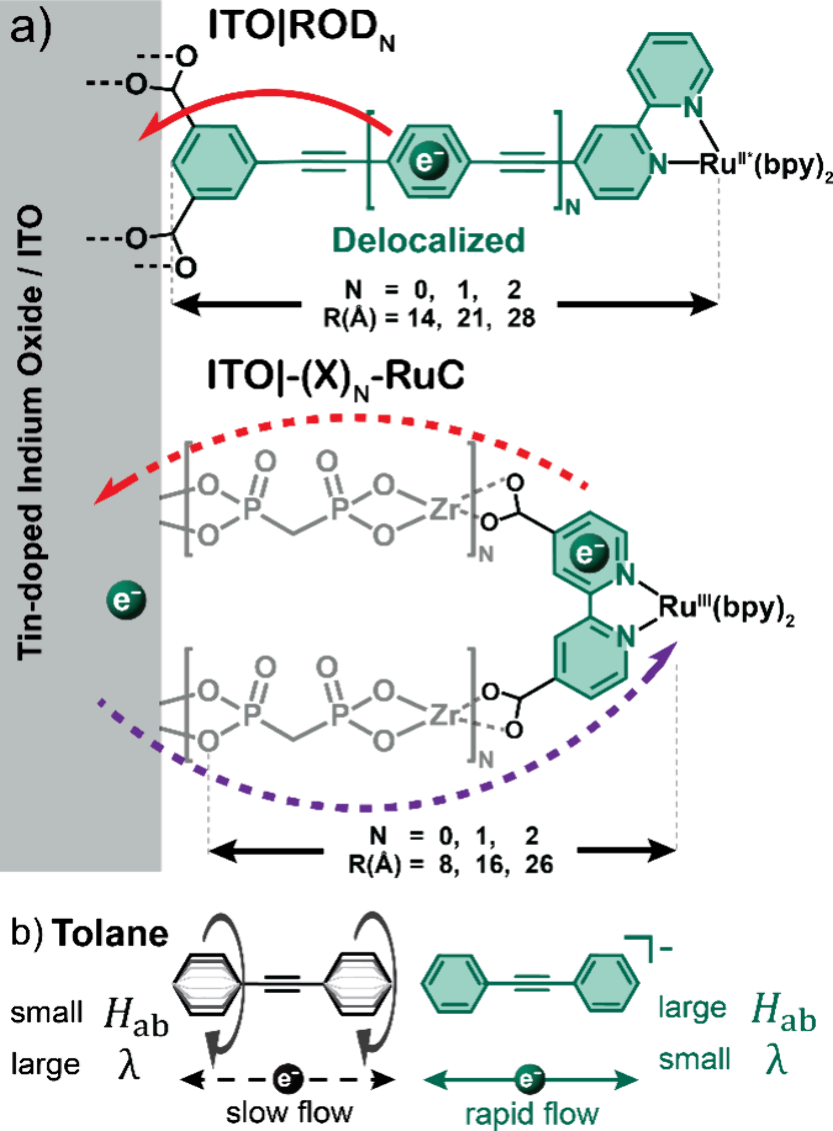
(a) Electron injection (red arrow), recombination (purple arrow), and length from the metal center to the carboxylate C atom for the rigid-rod and ionic-bridge complexes. (b) Molecular structures of neutral (white) and reduced (green) tolane. Enhanced electron flow occurs in the reduced state due to a larger electronic coupling matrix element *H*_ab_ and a smaller reorganization energy λ.

The DFT calculations provide the distance from the Ru to the midpoint of a line that connects the two carboxylic acid carbon atoms (Figure 8a; complete DFT-optimized structures are provided in Figure S6). In the MLCT excited state, the electron is present on the bipyridine ligand, and the electroabsorption study of related Ru polypyridyl complexes reveals this to be about 1 Å closer to the anchoring groups.^66^ Hence, the distances given are more appropriate for the unwanted recombination reaction. We emphasize and discuss further below that the true electron transfer distance depends on the degree of excited-state delocalization. As has been the subject of previous studies, the rigid rods can stand orthogonally or lean toward the oxide, and this surface orientation directly impacts the electron transfer distance.^45,67,68^ Since the actual electron transfer distances are unknown, the distances discussed herein are from the metal center to the anchoring group.

A simple functional model widely employed for gated electron transfer studies with PE bridge units is the molecule tolane (Figure 8b). Computational and experimental data indicate a significant 12 kcal/mol increase in the rotational barrier when tolane is oxidized or reduced by one electron.^34,35^ Analogous calculations of the one-electron-reduced form of ROD2 indicated a similar 10 kcal/mol increase in the rotational barrier between the pyridine ligand and the first phenyl group in the PE unit (Figure S7). This planar orientation promotes rapid electron flow, mainly attributed to strong coupling,^19^ although the smaller average bond distance is also expected to decrease the reorganization energy.^69^ Hence, the reduced tolane corresponds to the “open gate”, and the neutral form corresponds to the “closed gate”, which inhibits electron transfer. Note, however, that any degree of planarization, even that which gates between the extremes of strong (adiabatic) and weak (nonadiabatic) coupling, is expected to promote electron transfer equally in both directions. Vectorial electron transfer with these aromatic bridge units requires an asymmetry or a free energy change to drive electrons in one direction preferentially. The asymmetry in this study comes from the molecular orbitals involved in the interfacial electron transfer reactions and the applied potential that was used to tune −Δ*G*°. Excited-state injection involves the π* orbitals of the rigid-rod-bearing bipyridine and the ITO acceptor states, while recombination is from the ITO to Ru(III) d orbitals.

There is clear literature precedent that aromatic bridge units promote rapid electron transfer when the individual units planarize relative to a 90° rotation about the carbon–carbon bonds that link the bridge units together.^24,27−36^ The impact of this planarization can be so dramatic that some researchers have adopted xylyl, or other sterically hindered aromatics, to achieve a rigid bridge with more authentic through-space, nonadiabatic electron transfer.^64,65^ The one-electron reduction of the PE bridges promotes the planarization of the bridge units. MLCT excitation is also known to result in rapid picosecond coplanarization of aromatic substituents present at the 4 and 4′ positions of a bipyridine ligand.^70^ The regular increase in the excited-state absorption maximum as the number of PE units was increased from zero to two in this study suggests that the excited state is delocalized over the entire bridge, as observed previously. Below we discuss this interfacial structural gating within the confines of Marcus–Gerischer theory and the fundamental electron transfer parameters extracted from the kinetic data.

### Marcus–Gerischer Theory

The efficient long-distance vectorial electron transfer evident for the rigid-rod complexes, which is absent for the ionic-bridge complexes, could result from a small reorganization energy λ, an enhanced electronic coupling matrix element *H*_ab_, or changes to both λ and *H*_ab_. To identify the origins of this structural gating, the thermodynamic driving force was systematically tuned with an applied potential and the kinetic data were analyzed within the constructs of Marcus–Gerischer theory. Equation 4 is the semiclassical expression for the interfacial electron transfer rate constant:
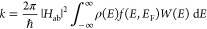
4where ρ(*E*) is the density of states of the conductor and *W*(*E*) is the classical Gaussian distribution of activation energies, given by
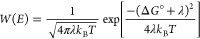
5The driving force for excited-state injection is −Δ*G°* = *e*(*E°*′* – *E*_F_), where *E°*′*(Ru^III/II*^) is the excited-state reduction potential. The Fermi energy, *E*_F_, was controlled with an applied potential *V*_app_ = *E*_F_.

**Figure 9:**
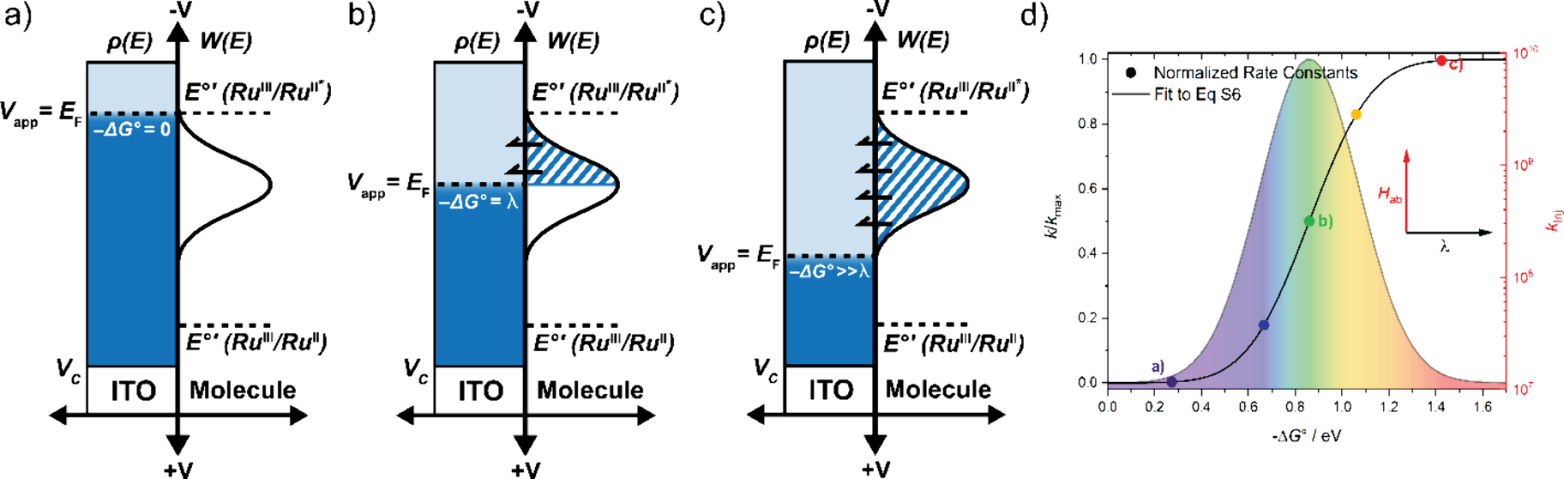
(a–c) Gerischer diagrams shown with potentiostatic control of the Fermi energy, *E*_F_, for a generic excited state *E°*′*(Ru^III/II*^) with (a) −Δ*G°* = 0, (b) −Δ*G°* = λ, and (c) −Δ*G°* = 2λ. (d) Plot of *k*/*k*_max_ versus −Δ*G°* with a fit to eq S6.

The Gerischer diagrams shown in Figure 9 depict three interfacial energetic conditions that correspond to −Δ*G°* = 0, −Δ*G°* = λ, and −Δ*G°* > 2λ. Marcus–Gerischer theory relates the maximum rate constant to the electronic coupling: *k*_max_ = . The free energy change that corresponds to *k*_max_/2 provides λ. A key assumption is that the electronic coupling is insensitive to the applied potential. Additional details of the Marcus–Gerischer approach are given in the Supporting Information and previously published works.^44,71,72^

**Figure 10:**
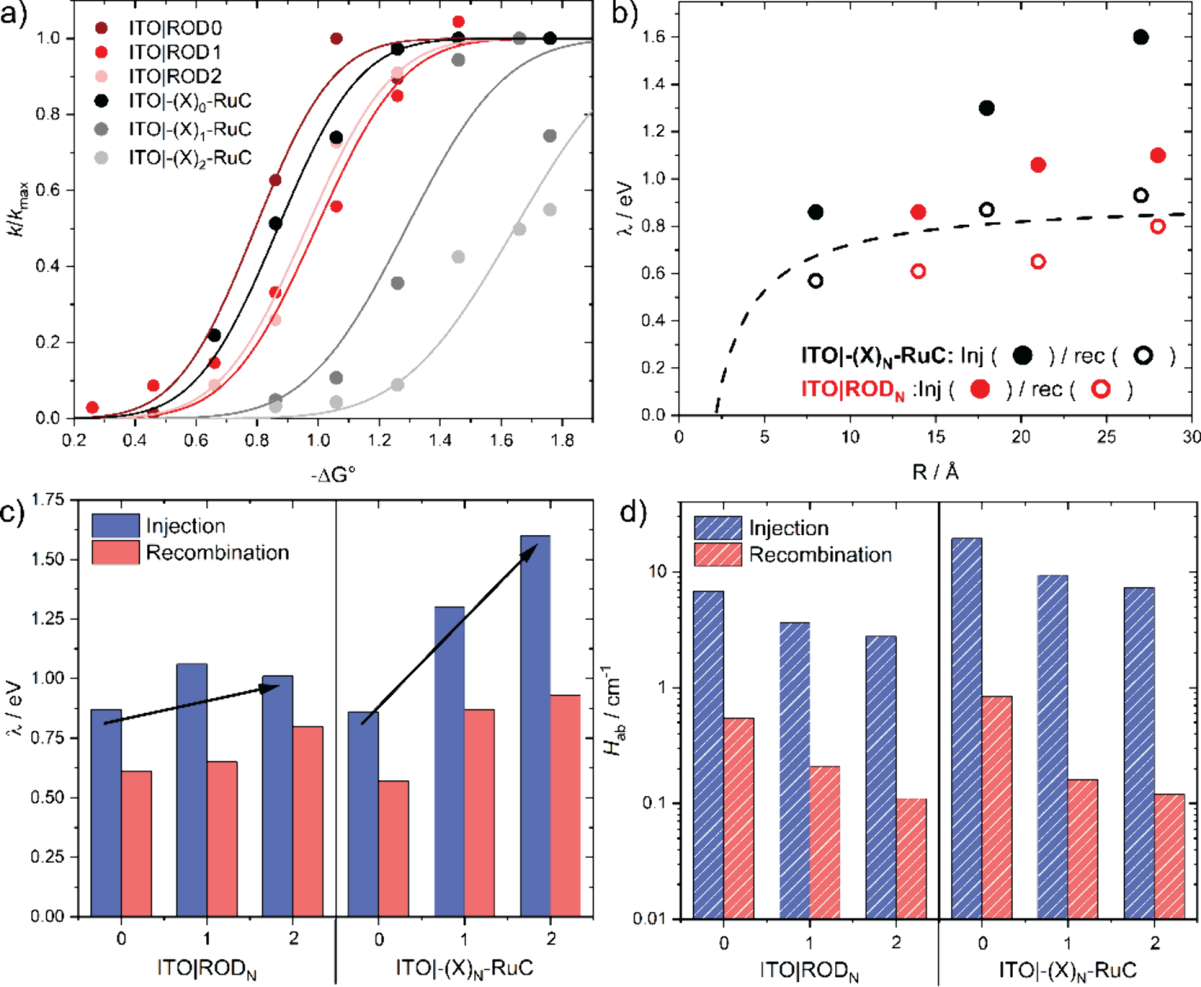
(a) Plot of *k*/*k*_max_ vs −Δ*G°* for the indicated ITO|rigid-rod and ITO|ionic-bridge samples. A satisfactory *k*_max_ was inaccessible for ITO|−(X)_2_–RuC, requiring estimation of the value (Figure S5). (b) Reorganization energies λ for electron injection and recombination, plotted as a function of the approximate electron transfer distance *R* for the indicated ITO|rigid-rod (red) and ITO|ionic-bridge (black) samples. Reorganization energies predicted by dielectric continuum theory are provided (dashed line). (c) Reorganization energies λ and (d) electronic coupling matrix elements *H*_ab_ for electron injection (blue) and recombination (red) plotted vs the indicated ITO|rigid-rod and ITO|ionic-bridge samples.

Experimentally, excited-state injection was slow when −Δ*G°* was small and increased to a saturation value *k*_max_ as the applied potential was more positive, corresponding to a more favorable free energy change. Such saturation behavior is consistent with Marcus–Gerischer theory and with previous studies.^44^ The extracted reorganization energies and couplings are shown in Figure 10 and are listed in Table 2.

Before the Marcus parameters are discussed in detail, two experimental details should be mentioned. First, the value of *k*_max_ was not obtained experimentally for ITO|−(X)_2_–RuC. At this interface, the electron transfer rate increased gradually with more favorable driving forces and did not reach a maximum, even when the potential was made so positive that ground-state oxidation of the metal center was evident. It was not possible to increase the driving force further, as complete oxidation resulted in a loss of the MLCT absorbance necessary to photoinitiate excited-state injection. Qualitatively, rate constants that increase gradually with −Δ*G°* represent a hallmark signature of a large reorganization energy. Still, a precise value remains elusive, and the estimates given were based on iterative modeling of the available data (Figure S5). Second, this analysis requires conversion of the applied potential to −Δ*G°* through knowledge of the excited-state reduction potential. The presence of two electronic excited states complicates such an analysis. Herein, the free energy present in the photoluminescent MLCT excited state was utilized, although the RR state is likely a weaker photoreductant that would effectively shift the abscissa in Figure 9d to smaller |−Δ*G°*| and hence would lower λ. Indeed, the apparent decrease in the reorganization energy for ITO|ROD2 relative to ITO|ROD1 may reflect excited-state injection from the RR state. However, the impact is expected to be small (<100 meV) since these two states are energetically proximate, as indicated by their quasi-equilibrium in kinetic competition with rapid microsecond relaxation to the ground state. We also emphasize that a slightly smaller λ for the PE-bridged complexes would only strengthen the conclusions drawn.

The reorganization energy for excited-state injection increased markedly with the number of ionic bridge units. In contrast, λ increased and stayed about the same when the number of PE bridge units increased. These small changes in λ are expected for a delocalization effect.^69^ As the electron delocalizes over additional bridge units, more bonds are indeed distorted, yet the average displacement of any individual bond decreases, resulting in a smaller overall distortion and hence reorganization energy. The total reorganization energy is the sum of the inner-sphere (λ_i_) and outer-sphere (λ_o_) contributions (eq 6):
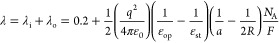
6Dielectric continuum theory relates the outer-sphere reorganization energy to the optical and static dielectric constants (ε_op_ and ε_st_), the molecular radius *a*, and the electron transfer distance *R*, as shown in eq 6. The inner sphere component for Ru(III/II) redox chemistry is known to be small, and a value λ_i_ = 0.2 eV^73^ was added to the λ_o_ values determined from eq 6 in Figure 10b (dashed line).

Delocalization may lead to enhanced electronic coupling, as was discussed for tolane. In addition to providing more significant overlap of the excited state and ITO wavefunctions, delocalization may decrease the electron transfer distance, resulting in a larger *H*_ab_. Interestingly, this expected increase in *H_ab_* due to excited state delocalization was not evident in the present study. Instead, *H*_ab_ was smaller for the PE bridges than for the ionic bridges. The origin(s) of this decreased coupling are unknown but likely emanate from increased triplet character in the RR state. Triplet states are known to be more localized and less strongly coupled than singlet excited states.^74,75^ Spin is generally a poor quantum number for the Ru heavy metal, where spin–orbit coupling mixes the singlet and triplet states. Previous cage-escape measurements^76,77^ and studies of transition metal complexes with covalently linked aromatic chromophores^78^ have revealed that a greater triplet character is present when the photoexcited electron is distant from the heavy metal center, as in the RR state.

Even though the coupling is relatively small for the rigid-rod state, the small λ allows efficient excited-state injection at >20 Å distances. In contrast, the large λ values for the ionic bridges resulted in inefficient excited-state injection even when the driving force was highly favorable. Indeed, activationless excited-state injection, expected when −Δ*G°* > 2 λ, could not be realized experimentally and would require over 3 eV of driving force for the complex with two ionic bridge units.

It is interesting to compare the Marcus parameters for excited-state injection to those for recombination. The *H*_ab_ values for excited-state injection were greater than 10-fold larger than those for recombination. This was qualitatively expected since excited-state injection occurs from the π* orbitals of the surface-anchored ligand to the oxide surface, while recombination occurs to the more distant Ru(III). The reorganization energy λ was larger for injection than for recombination, especially when ionic bridges were present. This behavior differs from the dye-sensitized TiO_2_ literature, where ultrafast excited-state injection is often associated with a small reorganization barrier.^59^ Many of the priorexcited-state injection measurements were performed with transition metal complexes that bear the 4,4′-(CO_2_H)_2_-2,2′-bipyridine (dcb) ligand. This ligand resides within the Helmholtz planes of the electric double layer, where a decreased solvent dielectric constant is expected to lower the kinetic barrier for electron transfer in the charge transfer excited state. Reorganization energies for long-distance interfacial electron transfer are not commonly reported at oxide interfaces and are instead better known for self-assembled monolayers (SAMs) with redox-active groups on gold surfaces.^79−81^

For recombination, λ varies over a relatively small range from 0.57 to 0.93 eV, consistent with values reported in the dye-sensitized TiO_2_ literature.^59^ The variation may reflect the location of the Ru(III) center in the electric double layer^41^ or that the recombination pathway includes the molecular orbitals of the bridge, as was shown by Berlinguette and Damrauer in comparative studies on semiconducting TiO_2_.^30,33^ In preliminary measurements, we used variable-temperature data to probe the role the bridge might play in interfacial electron transfer. An exciting aspect of aromatic bridge units is that increased temperature often leads to conformational changes that slow electron transfer, resulting in the appearance of negative activation energies.^82−91^ Kinetic data measured at −20 and +30 °C revealed that the excited-state injection rate measured after pulsed excitation of ITO|ROD2 was indeed inhibited at the higher temperature, while the charge recombination reaction displayed normal activated behavior (Figure S8).

Measurements at additional temperatures will be the focus of future work. However, these preliminary data provide no evidence that the PE bridge units are involved in the recombination reaction; this behavior enhances vectorial electron transfer.

We note that the gating mechanism described here supports the mathematical definition of vectorial electron transfer, as the yield of electrons transferred from the metal center to the ITO increased dramatically relative to those without the gating function. However, the kinetic representation of vectorial electron transfer is not supported. Previous authors have taken the ratio of the rate constants for the desired and undesired reactions as a “rectification ratio” and a measure of vectorial electron transfer.^59,92,93^ Since the rate constants for excited-state injection, *k*_2_, were largest for the ionic-bridge complexes and *k*_3_ was nearly insensitive to the nature of the bridge, the *k*_2_/*k*_3_ ratio is greater for the ionic bridges than for the PE bridges even though the yield of long-distance vectorial transfer was much higher. Slow nanosecond injection with a weak sensitivity to the number of PE bridge units has previously been noted for homoleptic star-shaped complexes, but the quantum yields were very low (ϕ_inj_ < 0.14).^94,95^ The high injection yields and structural gating reported herein result from different mechanisms for the injection and recombination processes. The data show that an enhanced vectorial electron transfer yield may occur in artificial photosynthetic assemblies, even when the rate constant for the desired reaction is small.

## Conclusion

The photophysical behavior of Ru(II) complexes anchored to insulating (ZrO_2_) and conductive (ITO) nanostructured metal oxide surfaces at variable positions within the electric double layer revealed a remarkably high quantum yield (∼1) for long-distance excited-state interfacial electron transfer when *p*-phenylene ethynylene (PE) bridge units were employed. The nearly quantitative yield results from excited-state delocalization on the PE units. After electron injection into ITO, rotation is restored, and the conjugated pathway is no longer available for the unwanted recombination reaction, thereby providing a structural gate for vectorial interfacial electron transfer. At the longest distances (>20 Å), such vectorial electron transfer is about 3-fold more efficient than in the case of bridge units that did not have the structural gating function. Marcus–Gerischer analysis of the kinetic data enabled the extraction of the reorganization energy and the electronic coupling matrix element for electron transfer. Electronic coupling with the conductive oxide surface was weak in all cases and indicated nonadiabatic electron transfer. Smaller coupling was evident for the PE bridge units relative to insulating ionic bridges, which indicated that coupling was not responsible for the high injection yields. Rather, an ∼0.7 eV smaller reorganization energy underlies the enhanced vectorial transfer through the conjugated aromatic bridge units.

These kinetic data are relevant to applications in dye-sensitized solar cells that utilize related Ru sensitizers. The dogma in this field is that there is stronger electronic coupling for excited-state injection into TiO_2_ relative to the recombination reaction; this behavior is expected based on the molecular orbitals involved in the transfer.^53^ Excited-state injection occurs from the π* orbitals of a diimine ligand, while recombination is to the more remote d orbitals of the metal center. This study reveals that this is not the case for sensitizers that have a gating function. The small reorganization energy for excited-state injection relative to the recombination reaction was unexpected and had little precedent in this literature. The data reported here indicate that a lower barrier for long-distance excited-state electron transfer can be realized through aromatic bridge units that support gated vectorial electron transfer.

## Supporting Information Available

Experimental methods for thin-film preparation, spectro-electrochemistry, nanosecond transient absorption spectroscopy, ultrafast transient absorption spectroscopy, density functional theory, and global kinetic analysis methods; 77 K steady-state and time-resolved photoluminescence; ultrafast transient absorption difference spectra and applied-potential-dependent kinetics of rigid rods and ionic bridges on ITO; tabulated applied-potential-dependent kinetic rate constants; description of Marcus–Gerischer kinetic analysis; DFT data including optimized rigid-rod structures, TD-DFT UV–vis absorption spectra, rigid-rod rotational barrier potential energy surfaces, and Z matrices for optimized rigid-rod geometries (PDF)
