# Supplementary material for: Structural Gating Enhances Long-Distance Light-Driven Interfacial Electron Transfer
Source: ACS Cent Sci. 2024 Nov 11;10(11):2132–44. doi: 10.1021/acscentsci.4c01106 (PMC11613339; doi:10.1021/acscentsci.4c01106)
Supplement: Supplementary file 1 — oc4c01106_si_001.pdf [file oc4c01106_si_001.pdf]

Supporting Information:

Structural Gating Enhances Long-Distance Light Driven Interfacial Electron Transfer

Quentin R. Loague[a], Marzieh Heidari[b], Hayden J. Mann[a], Evgeny O. Danilov[c], Felix N. Castellano[c], Elena Galoppini[b]*, Gerald J. Meyer[a]*

[a]*Department of Chemistry, University of North Carolina at Chapel Hill, Chapel Hill, NC, 27599, USA.*

[b]*Department of Chemistry, Rutgers University, 73 Warren Street, Newark, NJ, 07102, USA.*

[c]*Department of Chemistry, North Carolina State University, Raleigh, NC, 27695-8204, USA.*

*Corresponding authors: Elena Galoppini | Email: galoppin@rutgers.edu

Gerald J. Meyer | Email: gjmeyer@email.unc.edu

Table of Contents:

Experimental S2

Materials S2

Thin Film Preparation S2

Spectro-Electrochemistry S3

Nanosecond Transient Absorption Spectroscopy S4

Ultrafast Transient Absorption Spectroscopy (UFTA) S4

Quantum Yield Measurements S4

Variable Temperature Transient Absorption Spectroscopy S5

Density Functional Theory (DFT) S5

Global Analysis Methods S6

77 K Photoluminescence Data S8

Figure S1. 77 K Photoluminescence of Rigid-Rod Complexes S8

Transient Absorption Data S9

Figure S2. UFTA Difference Spectra on ITO S9

Figure S3. UFTA Applied Potential Dependent Kinetics S10

Figure S4. Normalized Applied Potential Dependent Kinetics S11

Table S1. Average kinetic rate constants for dye-sensitized ITO S12

Marcus – Gerischer Analysis **S12**
Figure S5. Regression of k_{max} for ITO|-(X)$_2$-RuC S13
Density Functional Theory (DFT) **S14**
Figure S6. DFT Optimized Structures for ROD 0,1,2 .... S14
Figure S7. Rotational Barrier Potential Energy Surface S15
Figure S8. Molecular Orbitals for Orthogonal Geometry S15
Figure S9. Molecular Orbitals for Planar Geometry S16
Z-Matrices for Optimized Ground-State Geometries S17
Variable Temperature Kinetic Data **S30**
Figure S10. Variable Temperature Kinetic Data for ITO|ROD2 .... S30
References **S31**

Experimental

Materials

Acetonitrile (CH_3CN, Burdick and Jackson, Honeywell, spectrochemical grade) and ethanol (99.5+ %, Acros Organics) were used as received. Hydroxypropyl cellulose (average M_w = 80 000, 20 mesh particle size), lithium perchlorate ($LiClO_4$, 99.999% trace metal), 2-[2-(2-Methoxyethoxy)ethoxy]acetic acid, methylene diphosphonic acid, and zirconyl chloride octahydrate (reagent grade, 98%) were obtained from Sigma-Aldrich. Nanoparticles of ITO (10% doping, 20 nm diameter) were purchased from Evonik Industries (VP ITO IR 5). Conductive FTO-coated (fluorine-doped tin oxide) glass substrates (15 Ω/sq) were purchased from Hartford Glass. The following complexes were available from previous studies: (ROD0)[1], (ROD1)[1], (ROD2)[1], and $[Ru^{II}(bpy)_2(dcb)](PF_6)_2$ (RuC),[2] where bpy is 2,2′-bipyridine and dcb is 4,4′-$(CO_2H)_2$-2,2′-bipyridine.

Thin Film Preparation

Mesoporous ITO thin films were prepared as previously described. Briefly, ITO nanoparticles were suspended in a 1:1 by weight ethanol:water solution with 20% ITO and 3% 2-[2-(2-Methoxyethoxy)ethoxy] acetic acid relative to ITO. For a 5 g suspension, this required 2.53 mL ethanol, 2 mL water, 1 g ITO, and 27 μL 2-[2-(2-Methoxyethoxy)ethoxy]acetic acid. To enhance dispersion, the suspension was sonicated with 10 second pulses by an ultrasonication horn

with care taken to avoid boiling. The suspension was centrifuged at 2000 rpm for 30 min and the supernatant was mixed with an equal volume of 10 wt% hydroxypropyl cellulose in ethanol under constant stirring. The resultant light blue viscous solution was doctor-bladed onto FTO-coated glass substrates with two layers of Scotch tape serving as a spacer. The materials were dried in air for 30 min and sintered at 450 °C for 30 min under an O_2 flow. The resulting materials were 3-4 μm thick (measured with a Bruker DektatXT profilometer) and were faintly yellow in color. Mesoporous thin films based on insulating ZrO_2 were prepared as previously described.[3]

The rigid-rods were anchored to the ITO films by overnight reaction in acetonitrile (ROD0 and ROD1, 5 mM). The equivalent surface attachment of ROD2 required heating the acetonitrile solution to 40 ℃ overnight (5mM solution). The surface coverages were quantified spectroscopically as described in the Results Section with a Varian Cary 60 spectrometer.

The $[Ru^{II}(bpy)_2(dcb)]^{2+}$ (RuC) was positioned at various distances from the ITO surface by a layer-by-layer assembly technique with methylenediphosphonic acid and Zr^{4+} cation ionic bridges.[4-7] Samples were prepared by first placing ITO thin films in a 0.1 M $HClO_4$ aqueous solution with 5 mM methylenediphosphonic acid overnight, followed by transfer to a 0.1 M $HClO_4$ aqueous solution with 6 mM $ZrOCl_2{\cdot}8\,H_2O$ for 2 hours. When needed a second ionic bridge was added by repeating this procedure. The ionic bridge functionalized ITO films were reacted with 5 mM RuC in methanol for 12 hours.

Spectro-Electrochemistry

All spectroelectrochemical and transient absorption spectroscopy experiments were performed in a standard three-electrode cell. The working electrode was an ITO film (sensitized with ROD_N or RuC) on conductive FTO-coated glass, the counter electrode was Pt mesh, and the reference electrode was a silver wire in 0.1 M $LiClO_4/CH_3CN$. The reference electrode was calibrated to the ferrocene reduction potential ($Fc^{+/0}$ = 630 mV vs NHE) in 0.1M *tert*-butylammonium perchlorate/acetonitrile.[8] All reduction potentials herein are referenced to NHE. The electrodes were assembled in a custom quartz cuvette with a fused 24/40 ground glass joint; the ITO film was positioned at a 45° angle. Electrical wires soldered to alligator clips were fed through a rubber septum to connect to working, counter, and reference electrodes. The septum was then used to seal the cuvette over the 24/40 joint. The cell was sparged with argon gas for 30 min prior to experiments.

Nanosecond Transient Absorption Spectroscopy

Transient absorption spectroscopy with nanosecond time resolution was performed in a standard three electrode cell. Pulsed excitation was accomplished using a RADIANT X30 tunable laser system: Q-switched, pulsed (10Hz) Nd:YAG laser (Quantel laser by Lumibird, Q-smart 450mJ) tripled to 355 nm, coupled with an OPO module (410-2500 nm tuning range). The laser fluence at the samples was 1-4 mJ/pulse (488 nm) at 5 Hz. A 150 W xenon arc lamp aligned perpendicular to the laser served as the probe beam. Two shutters placed between the arc lamp / laser and the sample were opened in 30 ms intervals to limit PMT fatigue. The probe light was focused onto the sample, collimated, and refocused onto an Oriel Cornerstone 260 monochromator optically coupled to a Hamamatsu R928 photomultiplier tube. The transient signal was recorded with a Teledyne Lecroy Wavesurfer 4024HD, 200 MHz digital oscilloscope (with variable bandwidth filters to improve the signal-to-noise ratio). The laser flashlamps, Q-switch, shutters, and oscilloscope trigger delays were controlled with a Berkley Nucleonics Corp. Model 577 Digital Delay Generator. The overall instrument response time was ~15 ns. Single-wavelength kinetics were generated as an average of 30-90 laser shots to achieve adequate signal-to-noise ratios. Transient absorption spectra were measured over a 385-800 nm range.

Ultrafast Transient Absorption Spectroscopy

Ultrafast TA measurements were performed at the North Carolina State University Imaging and Kinetic Spectroscopy (IMAKS) laboratory using a Helios transient absorption spectrometer (Ultrafast Systems).[9] Briefly, the beam from a 1 kHz Ti:sapphire Coherent Libra regenerative amplifier (4 mJ, 100 fs (fwhm) at 800 nm) was split into a pump and a probe beam. The pump beam was directed into an OperA Solo parametric amplifier (Coherent) to generate a 440 nm excitation (pump) source. The residual portion of the 800 nm beam was delayed in a 6.6 ns optical delay stage (Newport) before being focused into a CaF_2 crystal to generate a white light continuum (350-750 nm). The pump (800 μm diameter) and probe (200 μm) beams were focused onto the center of the surface functionalized ITO film, rotated 45° with respect to the probe beam.

Quantum Yield Measurements

The interfacial electron injection quantum yields were determined using comparative actinometry following previously reported procedures.[10] A ZrO_2 thin film sensitized with RuC served as an actinometer in nanosecond transient absorption spectroscopy measurements. The number of photons present in the measurement were quantified by monitoring the ground state bleach of RuC at 450 nm ($\Delta\varepsilon$ = -10,000 M^{-1} cm^{-1}). Analogous transient absorption measurements at the ground-excited state isosbestic points[1,2] of ITO|ROD_N and ITO|-$(X)_N$-RuC allowed quantification of the quantum yield for interfacial electron injection on a per photon basis.

The kinetic data collected for the quantum yield measurements were analyzed with a stretched exponential kinetic model, **Equation S1**. The change in amplitude obtained from the fit was utilized to calculate the quantum yield, as ~70% of the interfacial electron injection reaction has occurred on the timescale of the ultrafast transient absorption measurements. Prior interfacial electron injection quantum yield measurements via nanosecond transient absorption spectroscopy on sensitized TiO_2 are in remarkably good agreement[11].

Variable Temperature Transient Absorption Spectroscopy

Variable temperature ultrafast and nanosecond transient absorption spectroscopic data were obtained with a UniSoku CoolSpek (USP-203-B) liquid nitrogen cryostat. The samples were assembled in the standard three-electrode cell described above (the cryostat's sample chamber accommodates the dimensions of the custom quartz cuvette); cell was sparged with argon gas for 30 min prior to experiments. The cryostat has four quartz windows allowing both right angle (ns-TA) and colinear (UFTA) excitation. The samples were allowed to thermally equilibrate at each temperature for 20 minutes before beginning kinetic measurements.

Density Functional Theory (DFT)

Geometry optimizations of each rigid-rod complex was carried out to estimate the interfacial electron transfer distances.[1] All calculations were performed using Gaussian 16 with either the B3LYP functional (closed-shell calculations) or the UB3LYP functional (open-shell calculations).[12] The LANL2DZ with ECP basis set (obtained from Basis Set Exchange)[13] was used for Ru, while 6-311+G(d,p) was used for all other atoms. Each rigid-rod complex was optimized in the gas phase for both singlet and triplet ground-state electron configurations with a fixed dihedral angle of 0 or 90° between the between the pyridine ligand and the first phenyl group in

the PE unit. To model the solvation of each species in acetonitrile, each calculation specified an implicit solvent surface surrounding the rigid-rod complex using the Polarizable Continuum Model with the integral equation formalism variant (IEFPCM).[12] Occasionally, convergence was difficult to achieve in the triplet-state; in these cases, the option scf=xqc was specified in the route section to request quadratic convergence criteria. Single-point energy calculations (specified pop=(full,NO)) of the optimized geometries provided the molecular orbital energies relative to vacuum and produced the information necessary to visually render molecular orbitals (completed using IQmol v.3.1.4).

Time-dependent (TD) DFT calculations were performed on the optimized ground state geometries (with a dihedral angle of 0 and 90° between the between the pyridine ligand and the first phenyl group in the PE unit) for the singlet and triplet geometries. A total of sixty states were requested for each geometry. Only transition energies greater than 1.12 eV (~1100 nm) were considered, specified using the syntax td=(demin=1120) in the route section. TD-DFT calculations of the orthogonal geometry (dihedral angle of 90°) were utilized to characterize the MLCT excited state formed after light excitation. Subsequent planarization of the phenylene ethynylene bridge units gives rise to a new electronic state that is referred to as the RR state. To adequately model the rigid-rod, RR, state, TD-DFT calculations were performed on the optimized triplet geometry for the planar configuration (dihedral angle of 0°) which provided a method to approximate the molecular orbitals of the RR state formed after planarization of phenylene ethynylene bridge units. Examples of the LUMOs for each state are shown in **Figure 7b,** while complete molecular orbitals for the excited states of interest are provided in **Figure S8 and S9**.

To probe the barrier to rotation of a phenyl ring in the phenylene ethynylene bridge units, the dihedral angle of the unchelated ligand was scanned from 0 to 180°. Note that for ROD0, the dihedral angle is between the isophthalic acid and the pyridine. The rigid-rod ligands were optimized in the neutral and one-electron reduced states.

Global Analysis Methods

Full transient absorption spectra were collected for the sensitized ITO films at a series of applied potentials, V_{app}. Transient absorption measurements of the rigid-rods anchored to insulating ZrO_2 indicated rapid formation of a rigid-rod (RR) excited state in ~ 1ps, **Figure 3 (main text)**. Subsequent decay of the RR excited state was absent on the sub-6 ns timescale utilized in

the ultrafast transient absorption measurements, **Figure 3d (main text)**. When the rigid-rods were subsequently anchored to ITO films, amplitude changes in the transient absorption difference spectra were thus attributed to excited state injection. The resulting spectra were consistent with the formation of the interfacial charge separated state, ITO(e^-)|Ru^{III}.[1] To reduce the error associated with fitting ultrafast transient absorption kinetic data, a global analysis method was utilized. Here, the kinetic data from 1 ps to 6 ns was fit with a stretched exponential model (KWW) as a function of time and wavelength, λ, **Equation S1**. Here β is inversely related to the width of an underlying Levy distribution of rate constants, through which an average rate constant, $\bar{k},$ was determined from the first moment of this distribution, **Equation S2**.

Equation S1. $$\Delta A(t,\lambda) = A(\lambda)e^{-(kt)^{\beta}}$$

Equation S2. $$\bar{k} = \left[\frac{1}{k\beta}\Gamma\left(\frac{1}{\beta}\right)\right]^{-1}$$

Representative single wavelength kinetic data, with corresponding KWW fits, are provided as a function of V_{app} for each sample in **Figure S3**. To aid in qualitatively visualizing changes in the kinetic data as a function of V_{app}, the kinetic data in **Figure S3** were normalized from 0 to 1 using the preexponential factor, A, obtained from the global stretched exponential fits for a given wavelength, **Figure S4**. The applied potential dependent average kinetic rate constants are provided in **Table S1**.

77 K Photoluminescence Data

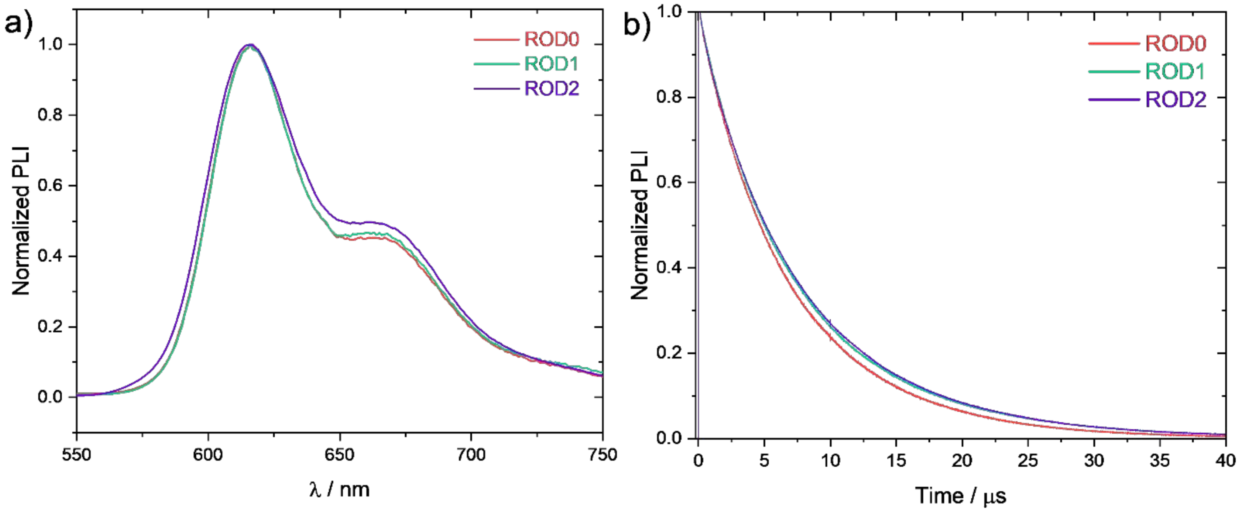

Figure S1. a) Normalized photoluminescence spectra of ZrO_2|RODx in MeTHF at 77 K. b) Time-resolved photoluminescence intensity decay kinetics, $\boldsymbol{\lambda_{obs} = 625\ \mathrm{nm}}$, of ZrO_2|RODx in MeTHF at 77 K.

Transient Absorption Data

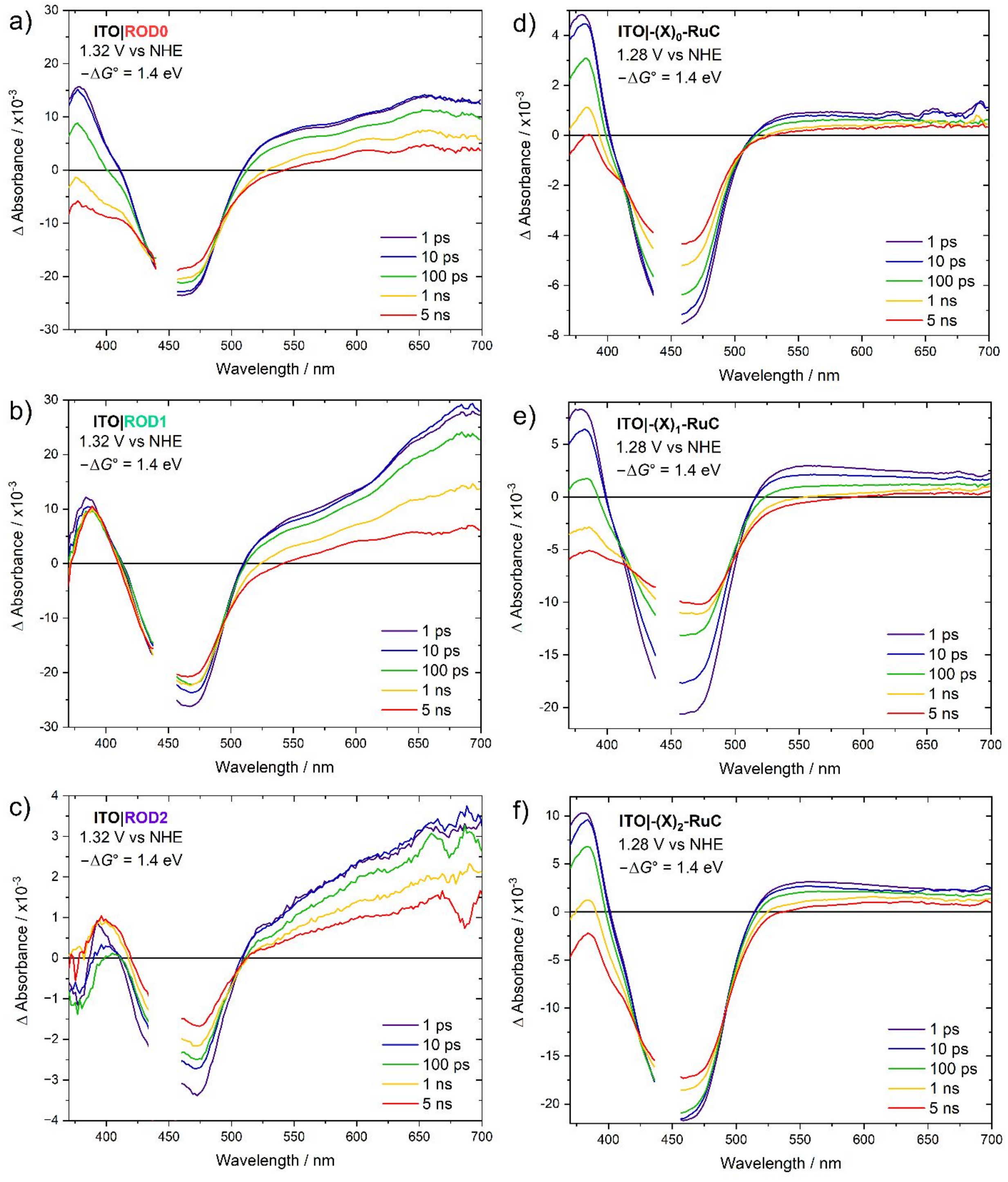

Figure S2. Transient absorption difference spectra measured at the indicated delay times after pulsed 440 nm laser excitation of (a) ITO|ROD0, (b) ITO|ROD1, (c) ITO|ROD2, (d) ITO|-$(X)_0$-RuC, (e) ITO|-$(X)_1$-RuC, and (f) ITO|-$(X)_2$-RuC in 0.1 M $LiClO_4/CH_3CN$ with the indicated applied potential that corresponds to $-\Delta G° = 1.4$ eV.

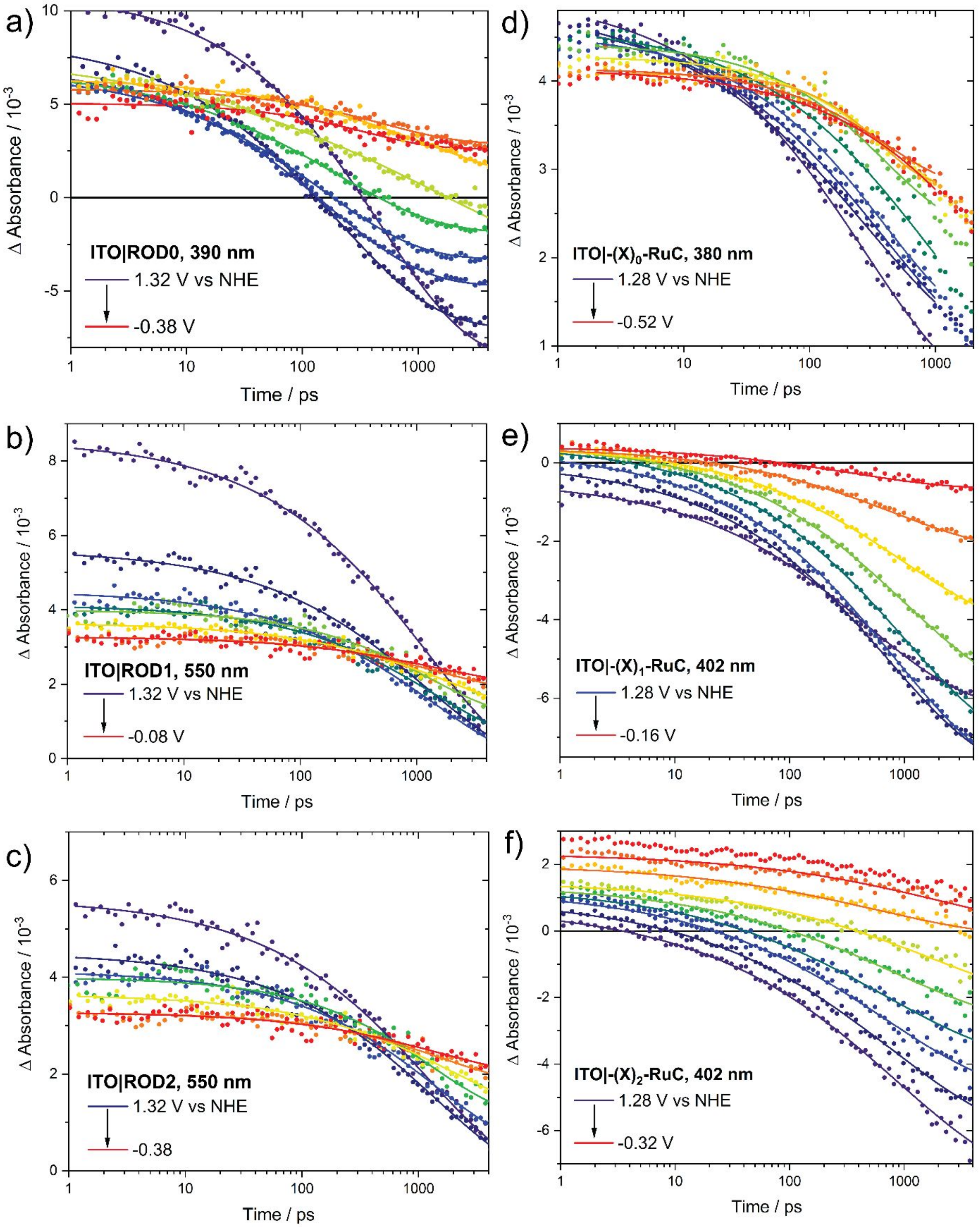

Figure S3. Transient absorption kinetics measured after pulsed 440 nm laser excitation (150 fs) of (a) ITO|ROD0, (b) ITO|ROD1, (c) ITO|ROD2, (d) ITO|-$(X)_0$-RuC, (e) ITO|-$(X)_1$-RuC, and (f) ITO|-$(X)_2$-RuC in 0.1 M $LiClO_4/CH_3CN$ at the indicated applied potentials.

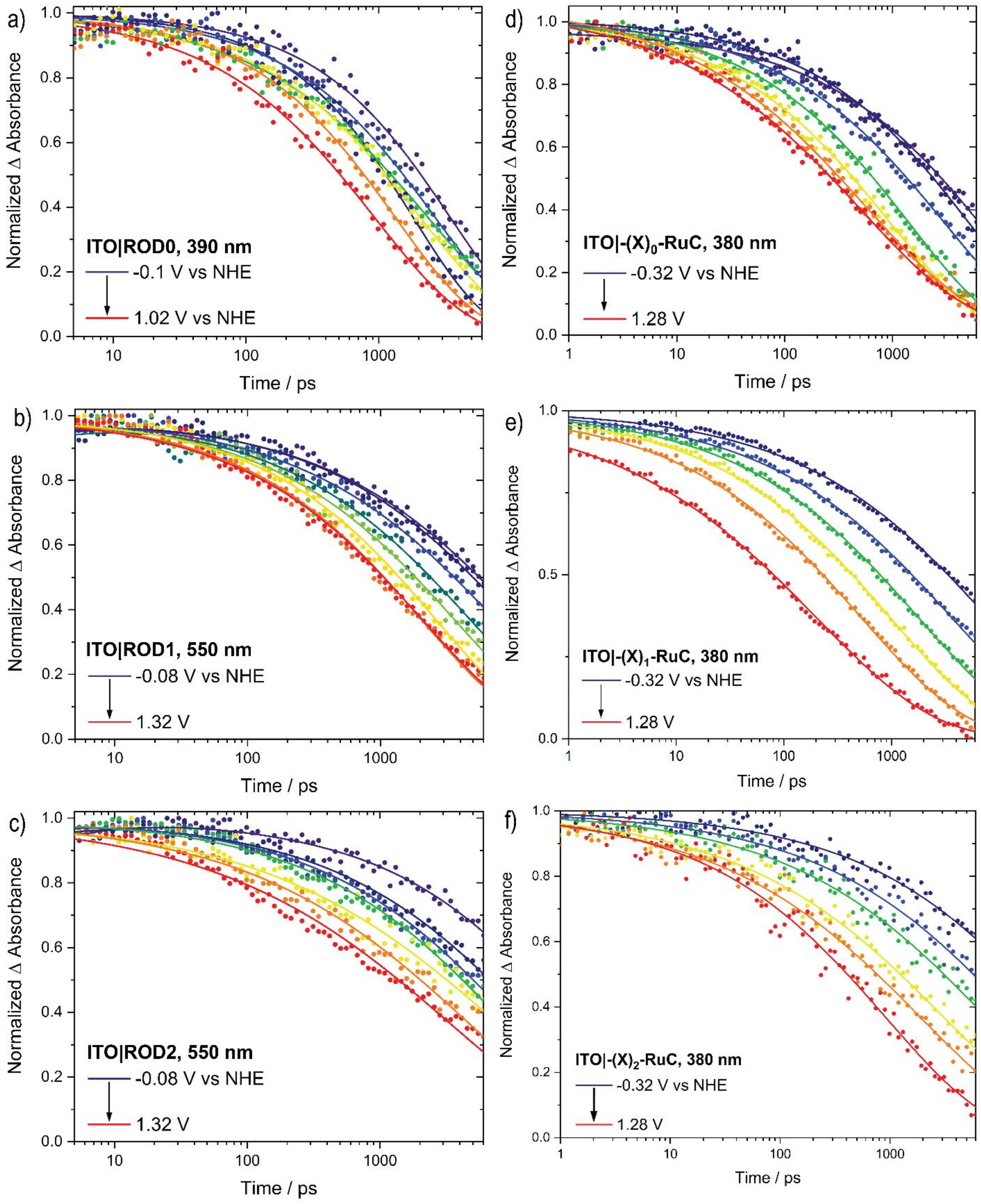

Figure S4. Normalized absorption change monitored after pulsed excitation (440 nm, 150 fs FWHM) of a) ITO|ROD0, b) ITO|ROD1, c) ITO|ROD2, d) ITO|-$(X)_0$-RuC, e) ITO|-$(X)_1$-RuC, f) ITO|-$(X)_2$-RuC in 0.1 M $LiClO_4/CH_3CN$ over the applied potential ranges indicate.

Table S1. Average kinetic rate constants for excited state electron injection measured in 0.1 M $LiClO_4/CH_3CN$.

	ITO|ROD0		ITO|ROD1		ITO|ROD2	
$-\Delta G°$	k / s^{-1}	β	k / s^{-1}	β	k / s^{-1}	β
**0.45**	3.5×10^{7}	0.46	4.8×10^{7}	0.54	3.5×10^{7}	0.52
**0.65**	5.6×10^{8}	0.52	8.1×10^{7}	0.49	7.1×10^{6}	0.47
**0.85**	1.6×10^{9}	0.58	1.8×10^{8}	0.57	2.1×10^{7}	0.44
**1.05**	2.5×10^{9}	0.62	3.1×10^{8}	0.55	5.8×10^{7}	0.46
**1.25**	2.3×10^{9}	0.57	4.7×10^{8}	0.56	7.3×10^{7}	0.48
**1.45**	N/A	N/A	5.7×10^{8}	0.55	7.9×10^{7}	0.50
**1.65**	N/A	N/A	5.5×10^{8}	0.54	8.0×10^{7}	0.47
**1.75**	N/A	N/A	N/A	N/A	N/A	N/A
**1.8**	N/A	N/A	N/A	N/A	N/A	N/A
	ITO|-$(X)_0$-RuC		ITO|-$(X)_1$-RuC		ITO|-$(X)_2$-RuC	
$-\Delta G°$	k / s^{-1}	β	k / s^{-1}	β	k / s^{-1}	β
**0.45**	2.5×10^{9}	0.69	N/A	N/A	N/A	N/A
**0.65**	4.1×10^{9}	0.65	N/A	N/A	N/A	N/A
**0.85**	7.6×10^{9}	0.53	N/A	N/A	N/A	N/A
**1.05**	1.3×10^{10}	0.62	2.2×10^{9}	0.58	1.6×10^{9}	0.53
**1.25**	1.8×10^{10}	0.52	4.8×10^{9}	0.54	2.1×10^{9}	0.5
**1.45**	1.5×10^{10}	0.55	1.6×10^{10}	0.61	4.4×10^{9}	0.46
**1.65**	N/A	N/A	4.2×10^{10}	0.62	2.1×10^{10}	0.48
**1.75**	N/A	N/A	4.4×10^{10}	0.54	2.5×10^{10}	0.46
**1.8**	N/A	N/A	3.4×10^{10}	0.53	2.7×10^{10}	0.47

Marcus – Gerischer Analysis

Interfacial electron transfer from the excited state to a conductive oxide (tin-doped indium oxide, ITO) was measured as a function of the driving force for excited state injection, $-\Delta G°$, to extract the reorganization energy, λ, and the electronic coupling matrix element, H_{ab}. In these kinetic studies, the Fermi energy was tuned with an applied potential, $V_{\mathrm{app}} = E_{\mathrm{F}}$, such that the driving force for excited state injection, $-\Delta G° = e(E^{\circ\prime *} - E_{\mathrm{F}})$, where $E^{\circ\prime}$ is the Ru polypyridyl complex's excited state reduction potential. $E^{\circ\prime *}$ for a given Ru sensitizer can be determined from **Equation S3**, where $E^{\circ\prime}$ is the ground state reduction potential of the $Ru^{III/II}$ couple and ΔG_{es} is the energy stored in the excited state.[11]

Equation S3. $$E^{\circ\prime *}\,(\mathrm{Ru^{III/II*}}) = E^{\circ\prime}(\mathrm{Ru^{III/II}}) + \Delta G_{\mathrm{es}}$$

The interfacial charge separation rate constants measured as a function of V_{app} were scaled to $-\Delta G°$ and analyzed with Marcus-Gerischer theory for nonadiabatic electron transfer, **Equation S4**, as previously described.[14-16] Here, the distribution of electron states in the ITO is described by $\rho(E)$ and their population is controlled by V_{app}. Ther Fermi-Dirac distribution is $f(E,E_{\mathrm{F}})$, the electronic coupling matrix element is H_{ab}, and the molecular donor states, $W(E)$, are described as a classical Gaussian distribution of activation energies, **Equation S5**.

Equation S4. $$k = \frac{2\pi}{\hbar}|H_{\mathrm{ab}}|^2\int_{-\infty}^{\infty}\rho(E)f(E,E_{\mathrm{F}})W(E)dE$$

Equation S5. $$W(E) = \frac{1}{\sqrt{4\pi\lambda k_{\mathrm{B}}T}}\exp\left(\frac{-(\Delta G°+\lambda)^2}{4\lambda k_{\mathrm{B}}T}\right)$$

The kinetic data were normalized to the maximum rate constant and fit to a modified error function to extract the reorganization energy, λ, for the charge separation reaction, **Equation S6**. The magnitude of $k_{\max}$ reports directly on H_{ab} through **Equation S7**, where ρ(E) = 0.45 eV^{-1} for ITO.[17]

Equation S6. $$\frac{k}{k_{\max}} = \frac{1}{2}\left[1-\mathrm{erf}\left(\frac{\Delta G^o+\lambda}{2\sqrt{\lambda \mathrm{k_B}T}}\right)\right]$$

Equation S7. $$k_{\max} = \frac{2\pi}{\hbar}{H_{\mathrm{ab}}}^2\rho(E)$$

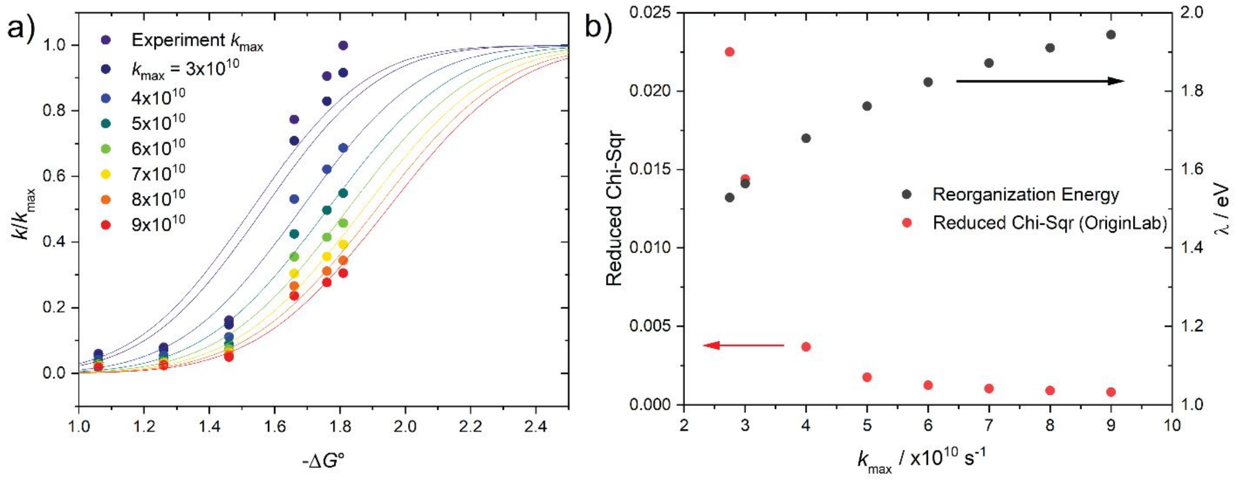

Figure S5. The $k_{\max}$ value was inaccessible experimentally for ITO|-(X)$_2$-RuC as the required applied potentials drove thermal oxidation of the Ru complex. In order to provide a more accurate estimate of λ, $k_{\max}$ was systematically varied to provide better a better fit to **Equation S6**. b) A plot of the extracted λ (black) and corresponding reduced chi-sqr values (red).

Density Functional Theory

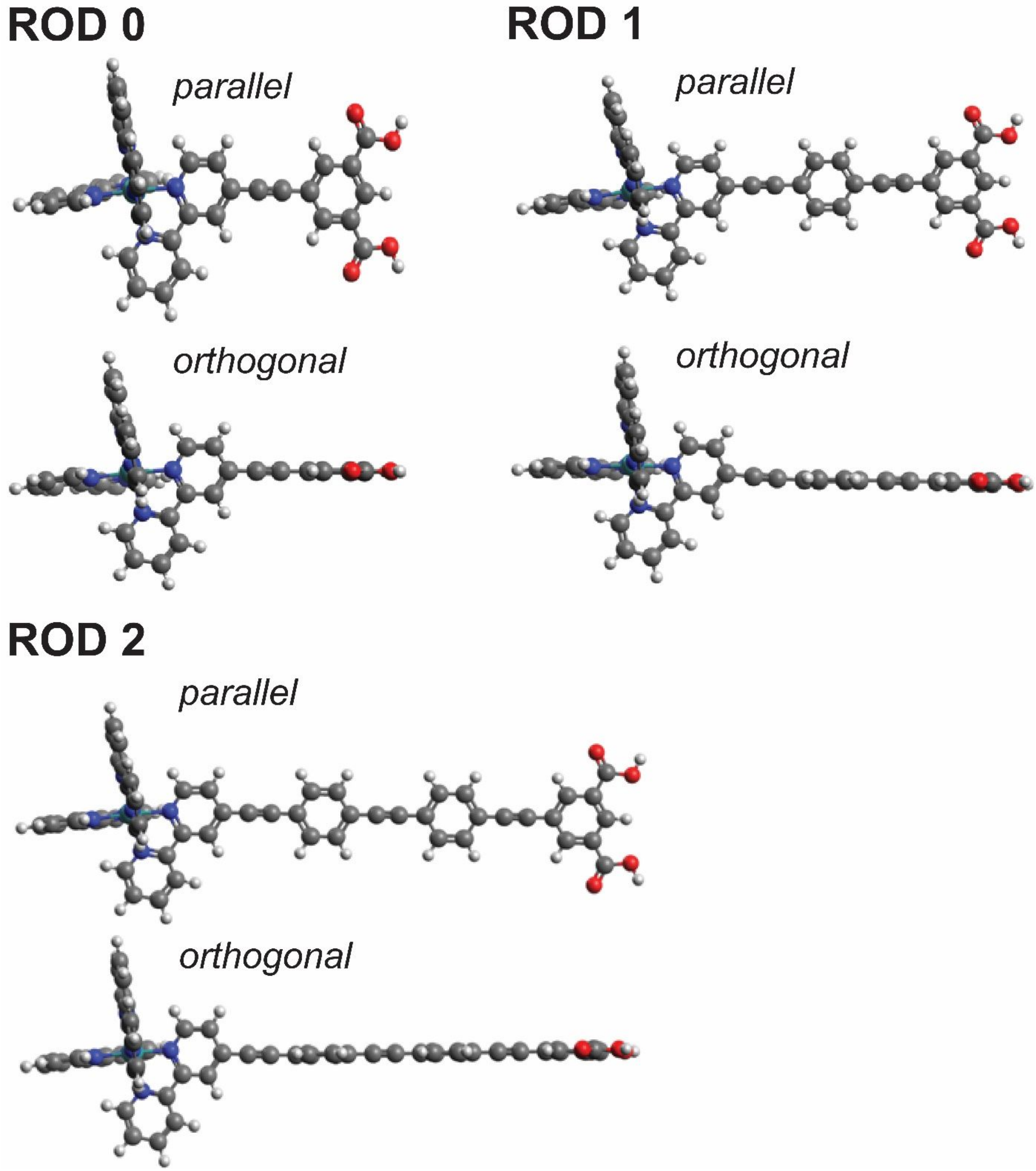

Figure S6. DFT optimized structures for when the bpy ligand is orthogonal (90°) and parallel (planar) with the PE bridge units (ROD1 and ROD2) or the isopthalic acid linker (ROD0).

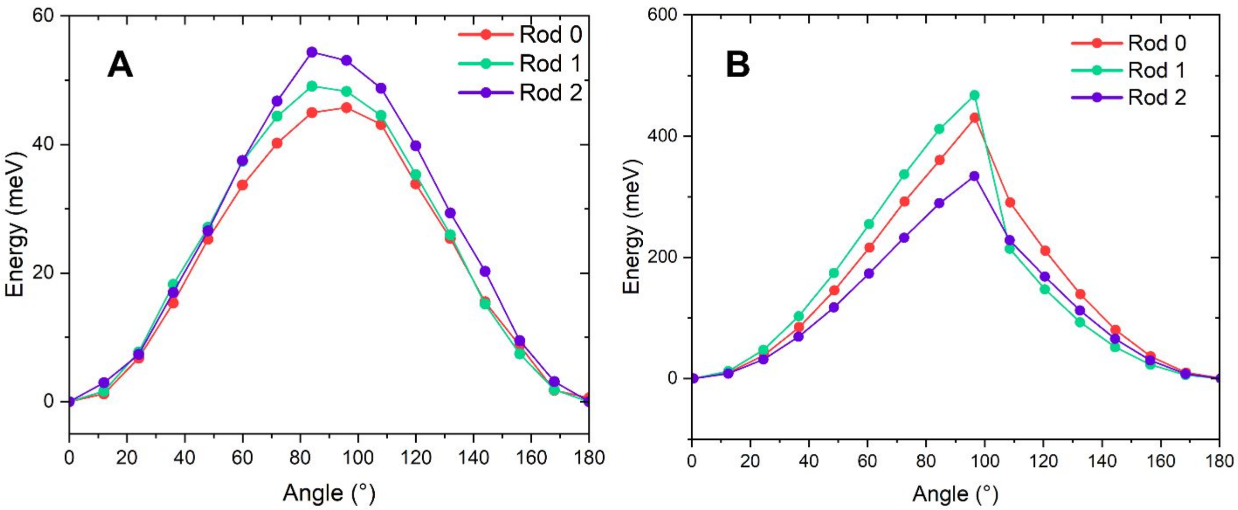

Figure S7. Theoretical potential energy surfaces (PES) mapping the rotational energy barrier of the bpy − rigid-rod dihedral angle for the A) neutral and B) one-electron reduced species.

ROD 2 (Orthogonal)
2.69 eV, f=0.3274

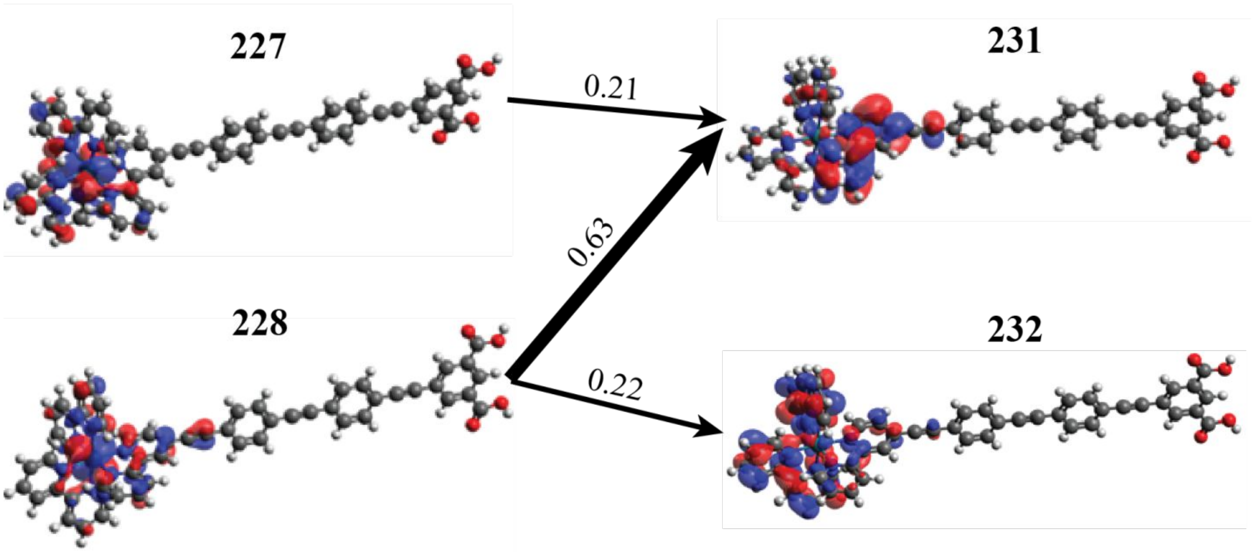

Figure S8. The molecular orbitals and corresponding transitions (with coefficients) for the 2.69 eV excited state, as determined from a TD-DFT calculation of the optimized orthogonal, ground state geometry.

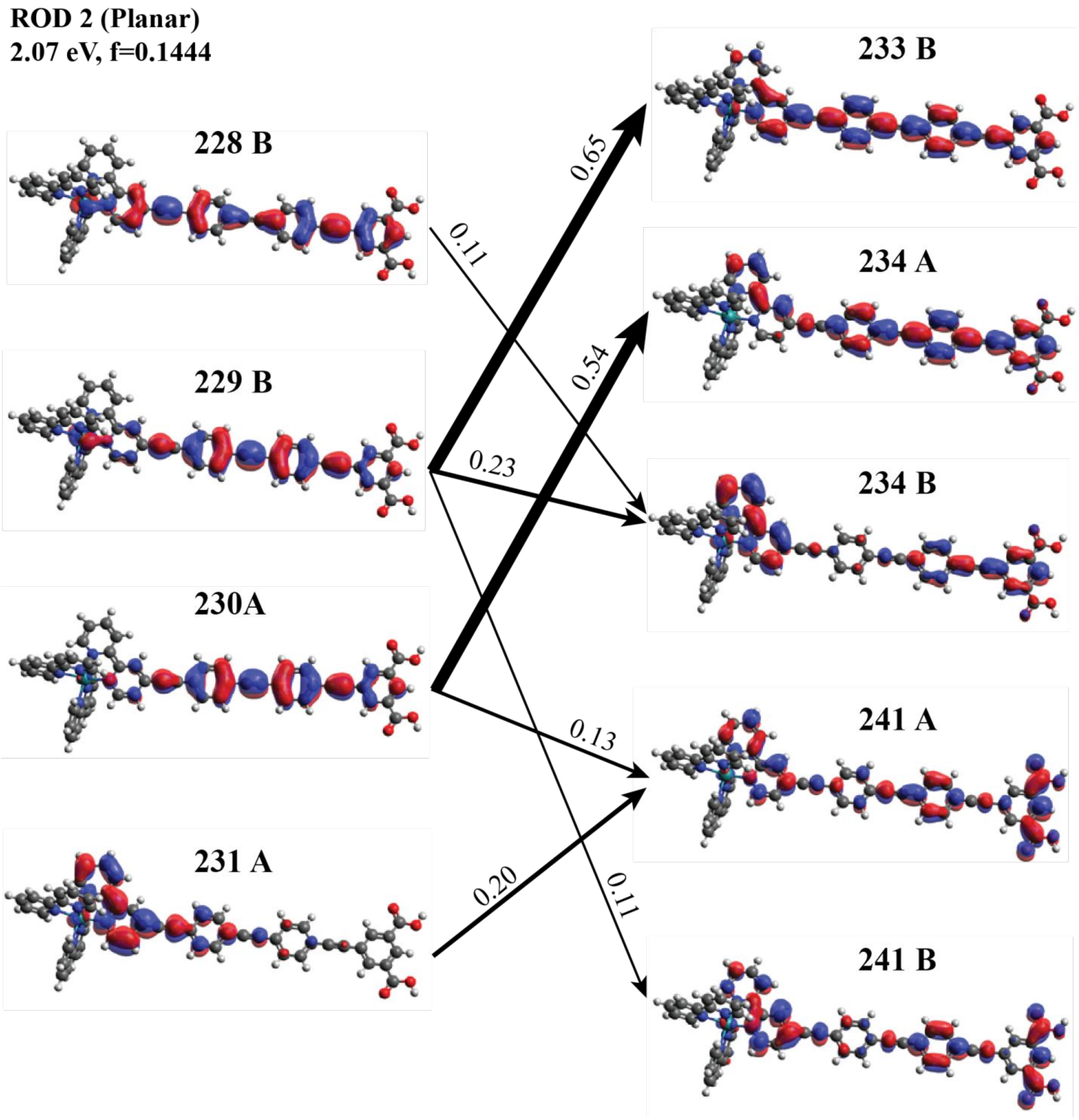

Figure S9. The molecular orbitals and corresponding transitions (with coefficients) for the 2.07 eV excited state, as determined from a TD-DFT calculation of the optimized planar, ground state geometry.

Z-Matrices for Optimized Ground-State Geometries

Rod 0 Singlet Parallel Geometry

C	-2.382374174985	-0.364700624154	-3.109230021413
H	-2.225926825414	0.700767202611	-3.078471997572
C	-2.287121106781	-1.079391778864	-4.297597752359
H	-2.050465181878	-0.564347432851	-5.216144769335
C	-2.498813692251	-2.459537276671	-4.276320414030
H	-2.428843764155	-3.042208109228	-5.182797298725
C	-2.800886340345	-3.078214051690	-3.065297571680
H	-2.963422999154	-4.144151785200	-3.036989337276
C	-2.888403049663	-2.317804909538	-1.893980850211
N	-2.677670402169	-0.959871170575	-1.925504991802
Ru(Iso=101.904)	-2.790330021800	0.024683477107	-0.066411714269
C	-3.482716938480	-2.404950722488	1.720949621132
H	-3.467321970490	-1.656450799492	2.495723555873
C	-3.771304220742	-3.735356785862	2.001455239725
H	-3.988050152515	-4.035394049576	3.015364215971
C	-3.775609890057	-4.661841594393	0.956573690867
H	-3.997921997871	-5.702024080844	1.142873607075
C	-3.489581023005	-4.225168097810	-0.334946475859
H	-3.492602493253	-4.931946238478	-1.149739394277
C	-3.203138782862	-2.875814417254	-0.569950226947
N	-3.201416891975	-1.971808793162	0.465526373062
C	-3.028045463282	2.924807134294	-1.124569291205
H	-4.080519243222	2.696876360296	-1.089401346093
C	-2.567711826549	4.169474628647	-1.536882876870
H	-3.272397681035	4.932930680632	-1.829414175161
C	-1.191723086924	4.407057698236	-1.561945527631
H	-0.803276528543	5.364704058120	-1.875370193872
C	-0.322682917747	3.389764839414	-1.174543849967
H	0.741697568565	3.563575991517	-1.187683399604
C	-0.831501541383	2.151167266366	-0.768365035242
N	-2.187328058455	1.927597350396	-0.747295847336
C	0.012584322008	-1.192130226643	0.466845164974
H	-0.569349393884	-2.045999983324	0.773008164153
C	1.395062263294	-1.223771650753	0.498517478032
H	1.907528666135	-2.112891331709	0.831954942605
C	2.132854921125	-0.088732206965	0.090626054842
C	1.393882140079	1.036964019978	-0.332882102608
H	1.930893909278	1.915978071835	-0.651587964289
C	0.002261005253	1.014100853032	-0.342396623248
N	-0.692404742099	-0.103514377505	0.059323779577
C	-5.708256290246	0.085829304878	-1.102607714547
H	-5.254079373652	-0.349912862256	-1.977126642307
C	-7.076154989158	0.323384440118	-1.035563095099
H	-7.707227019630	0.067666941404	-1.873051839062
C	-7.608421341910	0.890702652792	0.124231264737
H	-8.667436739232	1.084994043918	0.207069070181

C	-6.752627167346	1.202727661682	1.178239488628
H	-7.153798988157	1.638600444279	2.079646688692
C	-5.381449314007	0.947351203367	1.065241693116
N	-4.867479768396	0.388410884361	-0.080641451702
C	-2.133730500543	1.108849397638	2.756049717367
H	-1.137656756515	0.802488912637	2.482755692981
C	-2.401476724181	1.705836165169	3.982531683196
H	-1.599009287837	1.872006165194	4.684987097292
C	-3.712814569407	2.082416537093	4.280303516039
H	-3.951290823602	2.550704055193	5.223683930687
C	-4.713829184749	1.846798513575	3.340454639371
H	-5.729615809395	2.136127370811	3.559399577844
C	-4.398088925699	1.240917016320	2.119351994028
N	-3.103822310979	0.874996568338	1.835733458001
C	3.543687333149	-0.076391801370	0.105211669868
C	4.758985562548	-0.068409486024	0.118399629799
C	6.180319757153	-0.057086377981	0.133524693082
C	6.898608209880	-1.189552603355	0.562766972174
C	6.887532555868	1.087669560554	-0.280763109772
C	8.294446204330	-1.173902566098	0.576285871771
H	6.385194131056	-2.083195601187	0.886593300452
C	8.283239357932	1.097350615871	-0.265123215762
H	6.365456466336	1.972123956522	-0.615642712293
C	8.993243242329	-0.032032981299	0.163059101188
C	8.991763652458	-2.398726481072	1.039519733504
C	8.967201214360	2.334695606260	-0.714080250838
H	10.071183823888	-0.022620664509	0.174520995575
O	8.419795325256	-3.426897685628	1.411706681776
O	10.355469384235	-2.272248889365	1.012387737921
O	10.331898339117	2.234250062571	-0.657969466810
O	8.383878080117	3.352134784327	-1.099065328320
H	10.796661865041	-3.087088421132	1.320303707275
H	10.764298836821	3.057170848953	-0.956685639045

1 2 1.000 3 1.500 10 1.500
2 1 1.000
3 1 1.500 4 1.000 5 1.500
4 3 1.000
5 3 1.500 6 1.000 7 1.500
6 5 1.000
7 5 1.500 8 1.000 9 1.500
8 7 1.000
9 7 1.500 10 1.000 20 1.000
10 1 1.500 9 1.000 11 1.000
11 10 1.000 21 1.000 31 1.000 40 1.000 50 1.000 60 1.000
12 13 1.000 14 1.500 21 1.500
13 12 1.000
14 12 1.500 15 1.000 16 1.500
15 14 1.000
16 14 1.500 17 1.000 18 1.500
17 16 1.000

18 16 1.500 19 1.000 20 1.500
19 18 1.000
20 9 1.000 18 1.500 21 1.000
21 11 1.000 12 1.500 20 1.000
22 23 1.000 24 1.500 31 1.500
23 22 1.000
24 22 1.500 25 1.000 26 1.500
25 24 1.000
26 24 1.500 27 1.000 28 1.500
27 26 1.000
28 26 1.500 29 1.000 30 1.500
29 28 1.000
30 28 1.500 31 1.000 39 1.000
31 11 1.000 22 1.500 30 1.000
32 33 1.000 34 1.500 40 1.500
33 32 1.000
34 32 1.500 35 1.000 36 1.500
35 34 1.000
36 34 1.500 37 1.500 61 1.500
37 36 1.500 38 1.000 39 1.500
38 37 1.000
39 30 1.000 37 1.500 40 1.000
40 11 1.000 32 1.500 39 1.000
41 42 1.000 43 1.500 50 1.500
42 41 1.000
43 41 1.500 44 1.000 45 1.500
44 43 1.000
45 43 1.500 46 1.000 47 1.500
46 45 1.000
47 45 1.500 48 1.000 49 1.500
48 47 1.000
49 47 1.500 50 1.000 59 1.000
50 11 1.000 41 1.500 49 1.000
51 52 1.000 53 1.500 60 1.500
52 51 1.000
53 51 1.500 54 1.000 55 1.500
54 53 1.000
55 53 1.500 56 1.000 57 1.500
56 55 1.000
57 55 1.500 58 1.000 59 1.500
58 57 1.000
59 49 1.000 57 1.500 60 1.000
60 11 1.000 51 1.500 59 1.000
61 36 1.500 62 3.000
62 61 3.000 63 1.500
63 62 1.500 64 1.500 65 1.500
64 63 1.500 66 1.500 67 1.000
65 63 1.500 68 1.500 69 1.000
66 64 1.500 70 1.500 71 1.000
67 64 1.000
68 65 1.500 70 1.500 72 1.000

69 65 1.000
70 66 1.500 68 1.500 73 1.000
71 66 1.000 74 2.000 75 1.000
72 68 1.000 76 1.000 77 2.000
73 70 1.000
74 71 2.000
75 71 1.000 78 1.000
76 72 1.000 79 1.000
77 72 2.000
78 75 1.000
79 76 1.000

Rod 1 Singlet Parallel Geometry

C	-4.780227415063	0.395822112595	3.083038292301
H	-4.627067491697	-0.670330465314	3.062282510101
C	-4.692352277149	1.120129981702	4.266074334861
H	-4.465399753445	0.611864190386	5.190831207268
C	-4.898221210517	2.500908012256	4.231565255213
H	-4.833590438104	3.090859995853	5.133717611989
C	-5.187085835123	3.110518633453	3.012848486596
H	-5.344659248480	4.176884097137	2.973930310073
C	-5.267491179359	2.340577948053	1.847255596597
N	-5.063046956113	0.982031772162	1.891837587417
Ru(Iso=101.904)	-5.159299798544	-0.016695264065	0.040669717746
C	-5.828450413181	2.398623717817	-1.773159377622
H	-5.808593809912	1.643212248699	-2.541079321492
C	-6.109758143400	3.727437808885	-2.067814848541
H	-6.316448837625	4.019281547662	-3.086214617026
C	-6.119958818931	4.663134332521	-1.031135768361
H	-6.336883544041	5.702420939003	-1.228492288986
C	-5.847136838608	4.236739212239	0.266554674194
H	-5.854929889000	4.950448706613	1.075268371148
C	-5.567752193532	2.888436846663	0.515897173509
N	-5.559945213022	1.975407565334	-0.511566296412
C	-5.415154516079	-2.905996181640	1.122197523183
H	-6.466459904630	-2.675015223334	1.073875425889
C	-4.963404298304	-4.148084542803	1.550230185828
H	-5.673391557870	-4.906843962214	1.842149603650
C	-3.588176230001	-4.389104278209	1.591520418815
H	-3.205968155391	-5.345147437053	1.917382891289
C	-2.712342329197	-3.378079954542	1.204205128135
H	-1.648625409869	-3.554158093002	1.229455402658
C	-3.212340602270	-2.141024832145	0.781673439265
N	-4.567334658160	-1.914488972632	0.744907176828
C	-2.344503092907	1.188376853070	-0.474497953306
H	-2.920056007720	2.041698417984	-0.794578376149
C	-0.963285143415	1.215605009510	-0.492013672219
H	-0.445335847913	2.100534226505	-0.828080858992
C	-0.227822827257	0.081514191887	-0.065930940919
C	-0.980489222562	-1.038499431315	0.359934981186
H	-0.450591701541	-1.916786455253	0.692407273213
C	-2.370372909340	-1.010351554087	0.354286607893
N	-3.059716641834	0.105632284361	-0.064855340072
C	-8.091477894601	-0.064198000256	1.038428189011
H	-7.648404111949	0.380496899827	1.914188292183
C	-9.458476501857	-0.301699802416	0.955663266903
H	-10.100525807422	-0.036743459822	1.781874105258
C	-9.975603014684	-0.881217968948	-0.205028066075
H	-11.033524507288	-1.076030641772	-0.299821602545
C	-9.106100368710	-1.204561941936	-1.244222312399
H	-9.495406108685	-1.649639675224	-2.146358675061
C	-7.736365722615	-0.948450567221	-1.115750060893

N	-7.237215366129	-0.378032910138	0.031105139480
C	-4.466568784915	-1.124621689200	-2.761887314054
H	-3.474640356782	-0.815023544239	-2.477403689120
C	-4.718076138084	-1.733352636788	-3.986051965435
H	-3.906294565538	-1.905696950203	-4.676222857238
C	-6.025199150117	-2.113580162112	-4.297571254154
H	-6.250944556596	-2.590944144026	-5.239544610156
C	-7.038705714061	-1.869735749890	-3.373359025066
H	-8.051420804109	-2.161717970559	-3.602863293424
C	-6.739313994951	-1.252168450675	-2.153982180387
N	-5.449038265558	-0.882900736397	-1.856836166937
C	1.177277532357	0.065119148535	-0.065178550743
C	2.396563121563	0.057168392010	-0.065973687518
C	3.809591173199	0.050924071327	-0.067865646999
C	4.535099203850	1.187875229091	-0.498490471766
C	4.527777423015	-1.091352775807	0.360691535902
C	5.919539011877	1.181623532231	-0.499883128873
H	3.998891257953	2.066527550854	-0.828252611765
C	5.912139299113	-1.095649427214	0.358469922274
H	3.986009762230	-1.966087193171	0.691985618872
C	6.639079914446	0.040336752684	-0.071612439183
H	6.465393946607	2.053280235713	-0.829961949429
H	6.452509731626	-1.971305002455	0.687037569252
C	8.055827591644	0.034749885343	-0.073114800429
C	9.270357644472	0.029410758926	-0.074054684604
C	10.693961772304	0.022573273329	-0.074879456426
C	11.414570742154	1.154290613203	-0.500016677311
C	11.403735643543	-1.116226903634	0.349545555090
C	12.811851293398	1.145070396482	-0.499929437686
H	10.898751035546	2.043589402761	-0.831481899707
C	12.801019822915	-1.121010320593	0.347977890117
H	10.879416733873	-2.000313516396	0.681602597743
C	13.512027349456	0.008502749795	-0.076404281104
C	13.509907767454	2.367896011343	-0.959117607740
C	13.487232755564	-2.350695045776	0.806576284415
H	14.589697428572	0.003141263119	-0.077047587043
O	12.947443126719	3.397787298892	-1.342889957838
O	14.877885507439	2.248152808392	-0.917884579966
O	14.856270542390	-2.244873785765	0.763551539145
O	12.914895697179	-3.374787272814	1.191432459224
H	15.309297122751	3.067989469207	-1.225654832097
H	15.279755830551	-3.068991632357	1.070883719554

1 2 1.000 3 1.500 10 1.500
2 1 1.000
3 1 1.500 4 1.000 5 1.500
4 3 1.000
5 3 1.500 6 1.000 7 1.500
6 5 1.000
7 5 1.500 8 1.000 9 1.500
8 7 1.000

9 7 1.500 10 1.000 20 1.000
10 1 1.500 9 1.000 11 1.000
11 10 1.000 21 1.000 31 1.000 40 1.000 50 1.000 60 1.000
12 13 1.000 14 1.500 21 1.500
13 12 1.000
14 12 1.500 15 1.000 16 1.500
15 14 1.000
16 14 1.500 17 1.000 18 1.500
17 16 1.000
18 16 1.500 19 1.000 20 1.500
19 18 1.000
20 9 1.000 18 1.500 21 1.000
21 11 1.000 12 1.500 20 1.000
22 23 1.000 24 1.500 31 1.500
23 22 1.000
24 22 1.500 25 1.000 26 1.500
25 24 1.000
26 24 1.500 27 1.000 28 1.500
27 26 1.000
28 26 1.500 29 1.000 30 1.500
29 28 1.000
30 28 1.500 31 1.000 39 1.000
31 11 1.000 22 1.500 30 1.000
32 33 1.000 34 1.500 40 1.500
33 32 1.000
34 32 1.500 35 1.000 36 1.500
35 34 1.000
36 34 1.500 37 1.500 61 1.500
37 36 1.500 38 1.000 39 1.500
38 37 1.000
39 30 1.000 37 1.500 40 1.000
40 11 1.000 32 1.500 39 1.000
41 42 1.000 43 1.500 50 1.500
42 41 1.000
43 41 1.500 44 1.000 45 1.500
44 43 1.000
45 43 1.500 46 1.000 47 1.500
46 45 1.000
47 45 1.500 48 1.000 49 1.500
48 47 1.000
49 47 1.500 50 1.000 59 1.000
50 11 1.000 41 1.500 49 1.000
51 52 1.000 53 1.500 60 1.500
52 51 1.000
53 51 1.500 54 1.000 55 1.500
54 53 1.000
55 53 1.500 56 1.000 57 1.500
56 55 1.000
57 55 1.500 58 1.000 59 1.500
58 57 1.000
59 49 1.000 57 1.500 60 1.000

60 11 1.000 51 1.500 59 1.000
61 36 1.500 62 3.000
62 61 3.000 63 1.500
63 62 1.500 64 1.500 65 1.500
64 63 1.500 66 1.500 67 1.000
65 63 1.500 68 1.500 69 1.000
66 64 1.500 70 1.500 71 1.000
67 64 1.000
68 65 1.500 70 1.500 72 1.000
69 65 1.000
70 66 1.500 68 1.500 73 1.500
71 66 1.000
72 68 1.000
73 70 1.500 74 3.000
74 73 3.000 75 1.500
75 74 1.500 76 1.500 77 1.500
76 75 1.500 78 1.500 79 1.000
77 75 1.500 80 1.500 81 1.000
78 76 1.500 82 1.500 83 1.000
79 76 1.000
80 77 1.500 82 1.500 84 1.000
81 77 1.000
82 78 1.500 80 1.500 85 1.000
83 78 1.000 86 2.000 87 1.000
84 80 1.000 88 1.000 89 2.000
85 82 1.000
86 83 2.000
87 83 1.000 90 1.000
88 84 1.000 91 1.000
89 84 2.000
90 87 1.000
91 88 1.000

Rod 2 Singlet Parallel Geometry

C	-7.744384263564	-4.899708751510	6.032378463350
H	-7.218835382044	-5.827521311006	6.185676468471
C	-7.722987841722	-3.886091265579	6.983132457770
H	-7.169585947900	-4.021929576710	7.899820638258
C	-8.420612448417	-2.703517681889	6.728157748327
H	-8.420094104791	-1.897079950521	7.446319664680
C	-9.118132901500	-2.575974818159	5.529242074915
H	-9.657516759004	-1.665582037059	5.320078059507
C	-9.112935609545	-3.623917392724	4.602055080200
N	-8.423317794535	-4.784088577633	4.862641224966
Ru(Iso=101.904)	-8.465977629450	-6.233653477692	3.336983307201
C	-10.272930889556	-4.747484513163	1.313324427658
H	-10.112075893782	-5.641131471539	0.733449604153
C	-11.065261972319	-3.707613270923	0.841680566916
H	-11.536619081504	-3.788281511777	-0.125973625498
C	-11.236227608102	-2.572432136061	1.637021880789
H	-11.847276844930	-1.748916709528	1.298543006484
C	-10.607110696715	-2.516356297536	2.878458137372
H	-10.734325187939	-1.645574053231	3.502180586343
C	-9.817265228669	-3.587170969440	3.311703394077
N	-9.655021421317	-4.699815471840	2.521107090319
C	-7.457838563684	-8.640332954077	5.002649050093
H	-8.512330516611	-8.770403522358	5.181811925194
C	-6.517370802016	-9.510587896481	5.538976453485
H	-6.839366976364	-10.340304158468	6.149630678214
C	-5.163984979935	-9.290555582437	5.271376971580
H	-4.408928425074	-9.950700877331	5.671903246964
C	-4.798861921790	-8.206594015328	4.477458464207
H	-3.757185564349	-8.028854800724	4.262009339068
C	-5.780796314722	-7.353901934524	3.959470371335
N	-7.109435422732	-7.579677037502	4.229201798149
C	-6.419271825732	-4.404438576670	1.883106051541
H	-7.312451446299	-3.895853086300	1.558120885612
C	-5.169301738259	-3.955175070476	1.505684558455
H	-5.076584844873	-3.082396947214	0.877990890492
C	-4.006997543882	-4.639642737679	1.945559420587
C	-4.214986638731	-5.775049620193	2.765990589073
H	-3.352378737445	-6.317058998412	3.119484351843
C	-5.497271213989	-6.182747719187	3.112641498364
N	-6.608784338767	-5.498502363452	2.671070777848
C	-10.960957639331	-6.915351039416	5.042184756644
H	-10.569420883731	-6.129671580730	5.667065845078
C	-12.138144933662	-7.581291414579	5.362519418077
H	-12.681703196336	-7.311789821741	6.255256034963
C	-12.596324802503	-8.591829579707	4.514312903715
H	-13.508474472485	-9.125883510021	4.735473478798
C	-11.859788169696	-8.902246923775	3.373452861403
H	-12.206351636999	-9.678708058620	2.709730143407
C	-10.679201629922	-8.205665602565	3.091770129212

N	-10.236201824426	-7.213191816662	3.933573327355
C	-7.906251416399	-7.798143635060	0.730245049749
H	-7.064448775346	-7.127445301954	0.683007110820
C	-8.122045530945	-8.768936044300	-0.241037897427
H	-7.434488361090	-8.862470032122	-1.067809994695
C	-9.230751046663	-9.609920575932	-0.123771196770
H	-9.422845831795	-10.375134336039	-0.861137266413
C	-10.088960432424	-9.449837651605	0.961881532579
H	-10.946908443179	-10.095707222644	1.063476142277
C	-9.833503382720	-8.457405656424	1.914637544508
N	-8.738603399783	-7.635708623126	1.790253835154
C	-2.716866077853	-4.218362257062	1.589413811165
C	-1.596983263546	-3.846537639092	1.277828377244
C	-0.303825786307	-3.414205944472	0.916837025841
C	0.845168778071	-4.113001221401	1.363765816511
C	-0.125457183480	-2.270342536921	0.099389903341
C	2.112558004173	-3.687260652129	1.009772839275
H	0.724234857460	-4.987382937107	1.987880087915
C	1.142755253850	-1.845804661686	-0.253415475993
H	-0.992980215405	-1.727608665559	-0.249009103956
C	2.293761270062	-2.542934695604	0.192667378332
H	2.982322467139	-4.226544968105	1.355455618117
H	1.268413561184	-0.972600712433	-0.876713629088
C	3.588915531520	-2.108325938502	-0.167344346958
C	4.705232497372	-1.733858956740	-0.476615773174
C	6.005534542421	-1.297839862631	-0.835898495933
C	6.185319691022	-0.151824703069	-1.644114676845
C	7.150191900068	-1.999729068116	-0.393136552793
C	7.456975527692	0.274304373157	-1.994384543225
H	5.318367823649	0.393603041747	-1.989255964815
C	8.421386502850	-1.572931468706	-0.744112123449
H	7.026326771628	-2.877531069355	0.225214538716
C	8.601883980698	-0.426948422817	-1.551908446675
H	7.583465550291	1.151472646402	-2.612345562522
H	9.289631492457	-2.116446866232	-0.400528003808
C	9.904593449718	0.010066914938	-1.910665908102
C	11.018327439836	0.383410770240	-2.216880608954
C	12.325555615418	0.821364848291	-2.575961977288
C	12.503823770682	1.965982316183	-3.374858318515
C	13.462636191144	0.117368636246	-2.138235103899
C	13.787483611761	2.394196022833	-3.726122368960
H	11.651292310501	2.528177123327	-3.726738853989
C	14.744732788485	0.548597674639	-2.491487658210
H	13.359309785031	-0.765000879517	-1.523875888203
C	14.914312135941	1.688519690721	-3.286813623127
C	13.905257474179	3.605188686644	-4.568342499337
C	15.897881669848	-0.236614434030	-1.998168187313
H	15.903050334065	2.019723715796	-3.558332600303
O	12.953818535782	4.271474366719	-4.987802802147
O	15.210417503074	3.935136232645	-4.852841283074
O	17.108381173288	0.275960447769	-2.404599457230

O	15.816888445068	-1.248594535048	-1.294959525179
H	17.844240071759	-0.265939302838	-2.061606368266
H	15.249504669399	4.736616372233	-5.408618701923

1 2 1.000 3 1.500 10 1.500
2 1 1.000
3 1 1.500 4 1.000 5 1.500
4 3 1.000
5 3 1.500 6 1.000 7 1.500
6 5 1.000
7 5 1.500 8 1.000 9 1.500
8 7 1.000
9 7 1.500 10 1.000 20 1.000
10 1 1.500 9 1.000 11 1.000
11 10 1.000 21 1.000 31 1.000 40 1.000 50 1.000 60 1.000
12 13 1.000 14 1.500 21 1.500
13 12 1.000
14 12 1.500 15 1.000 16 1.500
15 14 1.000
16 14 1.500 17 1.000 18 1.500
17 16 1.000
18 16 1.500 19 1.000 20 1.500
19 18 1.000
20 9 1.000 18 1.500 21 1.000
21 11 1.000 12 1.500 20 1.000
22 23 1.000 24 1.500 31 1.500
23 22 1.000
24 22 1.500 25 1.000 26 1.500
25 24 1.000
26 24 1.500 27 1.000 28 1.500
27 26 1.000
28 26 1.500 29 1.000 30 1.500
29 28 1.000
30 28 1.500 31 1.000 39 1.000
31 11 1.000 22 1.500 30 1.000
32 33 1.000 34 1.500 40 1.500
33 32 1.000
34 32 1.500 35 1.000 36 1.500
35 34 1.000
36 34 1.500 37 1.500 61 1.500
37 36 1.500 38 1.000 39 1.500
38 37 1.000
39 30 1.000 37 1.500 40 1.000
40 11 1.000 32 1.500 39 1.000
41 42 1.000 43 1.500 50 1.500
42 41 1.000
43 41 1.500 44 1.000 45 1.500
44 43 1.000
45 43 1.500 46 1.000 47 1.500
46 45 1.000
47 45 1.500 48 1.000 49 1.500

48 47 1.000
49 47 1.500 50 1.000 59 1.000
50 11 1.000 41 1.500 49 1.000
51 52 1.000 53 1.500 60 1.500
52 51 1.000
53 51 1.500 54 1.000 55 1.500
54 53 1.000
55 53 1.500 56 1.000 57 1.500
56 55 1.000
57 55 1.500 58 1.000 59 1.500
58 57 1.000
59 49 1.000 57 1.500 60 1.000
60 11 1.000 51 1.500 59 1.000
61 36 1.500 62 3.000
62 61 3.000 63 1.500
63 62 1.500 64 1.500 65 1.500
64 63 1.500 66 1.500 67 1.000
65 63 1.500 68 1.500 69 1.000
66 64 1.500 70 1.500 71 1.000
67 64 1.000
68 65 1.500 70 1.500 72 1.000
69 65 1.000
70 66 1.500 68 1.500 73 1.500
71 66 1.000
72 68 1.000
73 70 1.500 74 3.000
74 73 3.000 75 1.500
75 74 1.500 76 1.500 77 1.500
76 75 1.500 78 1.500 79 1.000
77 75 1.500 80 1.500 81 1.000
78 76 1.500 82 1.500 83 1.000
79 76 1.000
80 77 1.500 82 1.500 84 1.000
81 77 1.000
82 78 1.500 80 1.500 85 1.500
83 78 1.000
84 80 1.000
85 82 1.500 86 3.000
86 85 3.000 87 1.500
87 86 1.500 88 1.500 89 1.500
88 87 1.500 90 1.500 91 1.000
89 87 1.500 92 1.500 93 1.000
90 88 1.500 94 1.500 95 1.000
91 88 1.000
92 89 1.500 94 1.500 96 1.000
93 89 1.000
94 90 1.500 92 1.500 97 1.000
95 90 1.000 98 2.000 99 2.000
96 92 1.000 100 1.000 101 2.000
97 94 1.000
98 95 2.000

99 95 2.000 103 2.000
100 96 1.000 102 1.000
101 96 2.000
102 100 1.000
103 99 2.000

Variable Temperature Kinetic Data

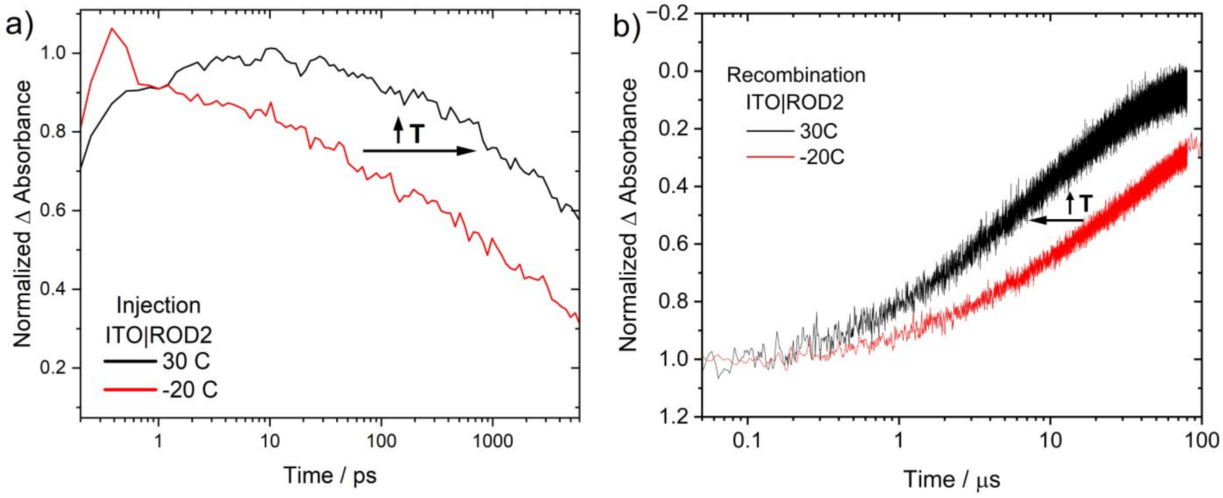

Figure S10. Transient absorption kinetics measured after pulsed 440 nm laser excitation [(a) 150 fs and (b) 10 ns] of ITO|ROD2 in 0.1 M $LiClO_4/CH_3CN$ at the indicated temperatures.

References

(1) Heidari, M.; Loague, Q.; Bangle, R. E.; Galoppini, E.; Meyer, G. J. Reorganization Energies for Interfacial Electron Transfer across Phenylene Ethynylene Rigid-Rod Bridges. *ACS Appl. Mater. Interfaces* **2022**, *14* (30), 35205-35214.
(2) Bangle, R. E.; Schneider, J.; Loague, Q.; Kessinger, M.; Müller, A. V.; Meyer, G. J. Free Energy Dependencies for Interfacial Electron Transfer from Tin-Doped Indium Oxide (ITO) to Molecular Photoredox Catalysts. *ECS J. Solid State Sci. Technol.* **2022**, *11* (2), 025003.
(3) Heimer, T. A.; D'Arcangelis, S. T.; Farzad, F.; Stipkala, J. M.; Meyer, G. J. An Acetylacetonate-Based Semiconductor−Sensitizer Linkage. *Inorg. Chem.* **1996**, *35* (18), 5319-5324.
(4) Wang, J. C.; Hill, S. P.; Dilbeck, T.; Ogunsolu, O. O.; Banerjee, T.; Hanson, K. Multimolecular assemblies on high surface area metal oxides and their role in interfacial energy and electron transfer. *Chem. Soc. Rev.* **2018**, *47* (1), 104-148.
(5) Dines, M. B.; DiGiacomo, P. M. Derivatized lamellar phosphates and phosphonates of M (IV) ions. *Inorg. Chem.* **1981**, *20* (1), 92-97.
(6) Putvinski, T.; Schilling, M. L.; Katz, H. E.; Chidsey, C. E.; Mujsce, A.; Emerson, A. Self-assembly of organic multilayers with polar order using zirconium phosphate bonding between layers. *Langmuir* **1990**, *6* (10), 1567-1571.
(7) Katz, H. E.; Scheller, G.; Putvinski, T. M.; Schilling, M. L.; Wilson, W. L.; Chidsey, C. E. D. Polar Orientation of Dyes in Robust Multilayers by Zirconium Phosphate-Phosphonate Interlayers. *Science* **1991**, *254* (5037), 1485-1487.
(8) Bard, A. J.; Faulkner, L. R. *Electrochemical Methods: Fundamentals and Applications*; Wiley, 2001.
(9) Garakyaraghi, S.; Danilov, E. O.; Mccusker, C. E.; Castellano, F. N. Transient Absorption Dynamics of Sterically Congested Cu(I) MLCT Excited States. *The Journal of Physical Chemistry A* **2015**, *119* (13), 3181-3193.
(10) Bergeron, B. V.; Kelly, C. A.; Meyer, G. J. Thin Film Actinometers for Transient Absorption Spectroscopy: Applications to Dye-Sensitized Solar Cells. *Langmuir* **2003**, *19* (20), 8389-8394.
(11) Wang, D.; Mendelsohn, R.; Galoppini, E.; Hoertz, P. G.; Carlisle, R. A.; Meyer, G. J. Excited state electron transfer from Ru(II) polypyridyl complexes anchored to nanocrystalline TiO_2 through rigid-rod linkers. *J. Phys. Chem. B* **2004**, *108* (43), 16642-16653.
(12) Frisch, M. J.; Trucks, G. W.; Schlegel, H. B.; Scuseria, G. E.; Robb, M. A.; Cheeseman, J. R.; Scalmani, G.; Barone, V.; Petersson, G. A.; Nakatsuji, H.; Li, X.; Caricato, M.; Marenich, A. V.; Bloino, J.; Janesko, B. G.; Gomperts, R.; Mennucci, B.; Hratchian, H. P.; Ortiz, J. V.; Izmaylov, A. F.; Sonnenberg, J. L.; Williams-Young, D.; Ding, F.; Lipparini, F.; Egidi, F.; Goings, J.; Peng, B.; Petrone, A.; Henderson, T.; Ranasinghe, D.; Zakrzewski, V. G.; Gao, J.; Rega, N.; Zheng, G.; Liang, W.; Hada, M.; Ehara, M.; Toyota, K.; Fukuda, R.; Hasegawa, J.; Ishida, M.; Nakajima, T.; Honda, Y.; Kitao, O.; Nakai, H.; Vreven, T.; Throssell, K.; Montgomery Jr, J. A.; Peralta, J. E.; Ogliaro, F.; Bearpark, M. J.; Heyd, J. J.; Brothers, E. N.; Kudin, K. N.; Staroverov, V. N.; Keith, T. A.; Kobayashi, R.; Normand, J.; Raghavachari, K.; Rendell, A. P.; Burant, J. C.; Iyengar, S. S.; Tomasi, J.; Cossi, M.; Millam, J. M.; Klene, M.; Adamo, C.; Cammi, R.; Ochterski, J. W.; Martin, R. L.; Morokuma, K.; Farkas, O.; Foresman, J. B.; Fox, D. J. Gaussian16. 2016.

(13) Pritchard, B. P.; Altarawy, D.; Didier, B.; Gibson, T. D.; Windus, T. L. New Basis Set Exchange: An Open, Up-to-Date Resource for the Molecular Sciences Community. *Journal of Chemical Information and Modeling* **2019**, *59* (11), 4814-4820.
(14) Farnum, B. H.; Morseth, Z. A.; Brennaman, M. K.; Papanikolas, J. M.; Meyer, T. J. Application of Degenerately Doped Metal Oxides in the Study of Photoinduced Interfacial Electron Transfer. *J. Phys. Chem. B* **2015**, *119* (24), 7698-7711.
(15) Gerischer, H. Electrochemical Techniques for the Study of Photosensitization. *Photochem. Photobiol.* **1972**, *16* (4), 243-260.
(16) Gerischer, H.; Willig, F. Reaction of excited dye molecules at electrodes. *Top. Curr. Chem.* **1976**, *61*, 31-84.
(17) Loague, Q.; Keller, N. D.; Müller, A. V.; Aramburu-Trošelj, B. M.; Bangle, R. E.; Schneider, J.; Sampaio, R. N.; Polo, A. S.; Meyer, G. J. Impact of Molecular Orientation on Lateral and Interfacial Electron Transfer at Oxide Interfaces. *ACS Appl. Mater. Interfaces* **2023**, *15* (28), 34249-34262.
